# Mode locking of hole spin coherences in CsPb(Cl, Br)_3_ perovskite nanocrystals

**DOI:** 10.1038/s41467-023-36165-0

**Published:** 2023-02-08

**Authors:** E. Kirstein, N. E. Kopteva, D. R. Yakovlev, E. A. Zhukov, E. V. Kolobkova, M. S. Kuznetsova, V. V. Belykh, I. A. Yugova, M. M. Glazov, M. Bayer, A. Greilich

**Affiliations:** 1 Experimentelle Physik 2, Technische Universität Dortmund, 44227 Dortmund, Germany; 2 Ioffe Institute, Russian Academy of Sciences, 194021 St. Petersburg, Russia; 3 P. N. Lebedev Physical Institute of the Russian Academy of Sciences, 119991 Moscow, Russia; 4 ITMO University, 199034 St. Petersburg, Russia; 5 St. Petersburg State Institute of Technology, 190013 St. Petersburg, Russia; 6 Spin Optics Laboratory, St. Petersburg State University, 198504 St. Petersburg, Russia

**Keywords:** Nanoscience and technology, Optics and photonics, Quantum information

## Abstract

The spin physics of perovskite nanocrystals with confined electrons or holes is attracting increasing attention, both for fundamental studies and spintronic applications. Here, stable ${{{{{{{\rm{CsPb}}}}}}}}{({{{{{{{{\rm{Cl}}}}}}}}}_{0.56}{{{{{{{{\rm{Br}}}}}}}}}_{0.44})}_{3}$ lead halide perovskite nanocrystals embedded in a fluorophosphate glass matrix are studied by time-resolved optical spectroscopy to unravel the coherent spin dynamics of holes and their interaction with nuclear spins of the ^207^Pb isotope. We demonstrate the spin mode locking effect provided by the synchronization of the Larmor precession of single hole spins in each nanocrystal in the ensemble that are excited periodically by a laser in an external magnetic field. The mode locking is enhanced by nuclei-induced frequency focusing. An ensemble spin dephasing time ${T}_{2}^{ * }$ of a nanosecond and a single hole spin coherence time of *T*_2_ = 13 ns are measured. The developed theoretical model accounting for the mode locking and nuclear focusing for randomly oriented nanocrystals with perovskite band structure describes the experimental data very well.

## Abstract (web summary)

Halide perovskites have a variety of attractive feature such as high quantum yield, and tunable optical properties, combined with easy fabrication. Here, Kirstein et al demonstrate spin-mode locking in CsPb(Cl0.56Br0.44)3 lead halide perovskite nanocrystals embedded in a fluorophosphate glass matrix, and a hole spin lifetime extending into the microsecond range.

## Introduction

Lead halide perovskite semiconductors are highly attractive due to their remarkable photovoltaic efficiency^1,2^, and are promising for optoelectronic^3,4^ and spintronic^4–7^ applications. Perovskite nanocrystals (NCs) have recently extended the wide class of semiconductor NCs grown by colloidal synthesis^8–13^. They show a remarkable quantum yield of up to 90% even for bare NCs, as surface states do not act detrimentally on the exciton emission efficiency. For inorganic CsPb*X*_3_ (*X* = I, Br, or Cl) NCs, the band gap can be tuned from the infrared up to the ultraviolet by mixing the halogen composition and by changing NC size, varying the quantum confinement of charge carriers. Additionally, the exciton fine structure can be adjusted by the NC shape^14–17^.

In addition to the high quantum yield and tunable optical properties, the simple fabrication makes lead halide perovskite NCs interesting for applications. As far as spintronics is concerned, only quite a few studies have been performed so far. Neutral and charged excitons in single NCs were identified by their Zeeman splitting in magnetic field^18,19^. For NC ensembles, negatively charged excitons (trions) and dark excitons were identified in strong magnetic fields of 30 T^20^. Optical orientation, optical alignment and anisotropic exciton Zeeman splitting were observed^21^. The coherent spin dynamics of electrons and holes in CsPbBr_3_ NCs^22,23^ were explored, and the picosecond spin dynamics of carriers in CsPbI_3_ NCs were reported^24^. Despite the progress in crystal growth, perovskite NCs still suffer from insufficient long-term stability of optical properties. A promising approach here is to synthesize NCs embedded in glass, providing protection by encapsulation^25,26^.

As a result, perovskite NCs in a glass matrix may be suitable for quantum technologies. For reference, one may compare NCs with the established system of self-assembled (In,Ga)As/GaAs quantum dots (QDs) singly charged with an electron or a hole. Using all-optical approaches one can orient and manipulate the spins of the charge carriers on the picosecond timescale at high operation frequencies^27,28^. There are two complementary concepts: the first one uses a single spin in a QD for establishing a quantum bit, the second one exploits a QD ensemble which may be suited for a quantum memory with sufficiently strong light-matter interaction. Here, we focus on a NC ensemble, that provides information about the average spin properties and their dispersion due to inhomogeneity. The inhomogeneity obstacle, however, can be overcome by applying a periodic laser excitation that synchronizes the electron spin Larmor precession in different NCs subject to a transverse magnetic field. This spin mode locking (SML) effect, initially discovered in singly charged (In,Ga)As QDs, allows one to uncover the spin coherence of individual carriers, which is typically prevented due to the faster spin dephasing in the ensemble^29–31^. The interaction of resident carrier spins with the surrounding nuclear bath provides further flexibility with potential memory times up to hours and hyperfine interaction fields up to a few Tesla^32,33^. This collective phenomenon of spin dynamics homogenization has been demonstrated so far only for (In,Ga)As QDs.

In this paper we demonstrate the SML effect for holes in a totally different class of QDs, namely perovskite ${{{{{{{\rm{CsPb}}}}}}}}{({{{{{{{{\rm{Cl}}}}}}}}}_{0.56}{{{{{{{{\rm{Br}}}}}}}}}_{0.44})}_{3}$ NCs synthesized in a glass matrix. We provide detailed information on the spin dynamics of confined holes correlated with the appearance of spin mode locking. We measure the *g*-factor, spread of *g*-factors, longitudinal spin relaxation time *T*_1_, transversal spin coherence time *T*_2_, and inhomogeneous spin dephasing time ${T}_{2}^{ * }$. Exploiting the hyperfine interaction with nuclear spins, we implement dynamic nuclear polarization and optically-detected nuclear magnetic resonance (ODNMR) to identify the involved nuclear isotopes. A theoretical model of the SML in perovskite structures is developed to account for the inverted band structure and for the dominating role of the hole-nuclear interaction, compared to self-assembled QDs. As an additional challenge, the random orientation of NCs in the ensemble is considered.

## Results

**Fig. 1:**
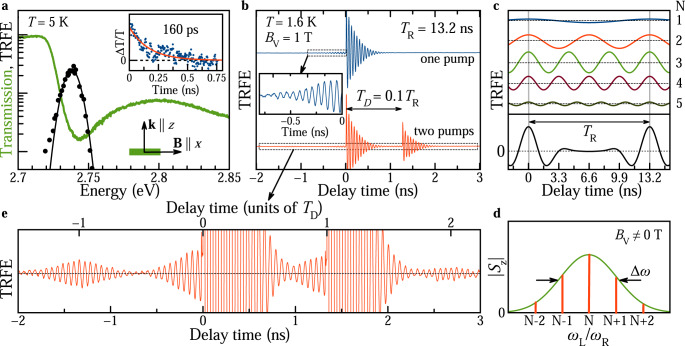
Spin mode locking in CsPb(Cl, Br)_3_ NCs. **a** Transmission spectrum of ${{{{{{{\rm{CsPb}}}}}}}}{({{{{{{{{\rm{Cl}}}}}}}}}_{0.56}{{{{{{{{\rm{Br}}}}}}}}}_{0.44})}_{3}$ NCs (green line). Black dots show spectral profile of the Faraday ellipticity amplitude measured at zero pump-probe time delay fitted by a Gaussian function (black line). *T* = 5 K. Inset shows time-resolved differential transmission (dots) with a monoexponential fit (red line) providing the exciton lifetime of 160 ps. **b** Time-resolved Faraday ellipticity (TRFE) measured at the photon energy of 2.737 eV with 1.5 ps laser pulses. Blue curve shows data for the one-pump protocol with a repetition period of *T*_R_ = 13.2 ns using the pump power *P*_pu_ = 25 mW. Inset shows zoom of signal before the arrival of the pump pulse, indicated by the box. Red curve is TRFE for the two-pump protocol (*T*_D_ = 0.1*T*_R_ = 1.32 ns) using the pump powers *P*_pu,1_ = 25 mW and *P*_pu,2_ = 13 mW. *T* = 1.6 K and *B*_V_ = 1 T. **c** Scheme shows spins precessing at five lowest PSC mode frequencies. Black curve at the bottom shows sum signal of these modes, weighted assuming an ensemble with a Gaussian distribution of amplitudes, as shown in panel d by the red vertical lines. **d** Illustration of the pumped carrier spin polarizations in inhomogeneous NC ensemble subject to a magnetic field. Green line shows the distribution of carrier precession frequencies caused by dispersion of the Larmor frequency. It is modeled by Gaussian with width Δ*ω*. PSC modes for the one-pump protocol, fulfilling the condition *ω*_L_ = *Nω*_R_ = 2*πN*/*T*_R_, are shown by the red vertical lines. **e** Zoom of the TRFE signal in the two-pumps protocol from **b**, indicated by the box. Upper scale gives delay in units of separation, *T*_D_, between two pumps. The bursts in the signal are associated with the electron spins with Larmor frequencies that are commensurate with the frequency: *ω*_D_ = 2*πM*/*T*_D_.

The optical properties of ${{{{{{{\rm{CsPb}}}}}}}}{({{{{{{{{\rm{Cl}}}}}}}}}_{0.56}{{{{{{{{\rm{Br}}}}}}}}}_{0.44})}_{3}$ nanocrystals embedded in a fluorophosphate glass are shown in Fig. 1a. At a cryogenic temperature of *T* = 5 K the transmission spectrum of the NCs, with about 8 nm size, shows a pronounced exciton resonance at 2.743 eV, which is broadened due to NC size dispersion. The exciton resonance can be also traced by the spectral dependence of the time-resolved Faraday ellipticity (TRFE) amplitude. The exciton population dynamics is measured by time-resolved differential transmission (Δ*T*/*T*), showing a decay time of 160 ps, which corresponds to the exciton lifetime (inset in Fig. 1a).

To study the coherent spin dynamics of resident carriers we use the pump-probe technique with spin-sensitive detection of the Faraday ellipticity, whose potential has been approved for lead halide perovskite crystals^34–36^, polycrystalline films^23,37,38^, and CsPbBr_3_ NCs^22,23^. The pump laser pulses are circularly polarized and, according to the selection rules, generate spin polarization of electrons and holes along the light **k** vector. The oriented carrier spins precess at the Larmor frequency around the magnetic field applied in the Voigt geometry *B*_V_ (**B**⊥**k**). The coherent spin dynamics is then detected through the Faraday ellipticity of the linearly-polarized probe pulses.

**Fig. 2:**
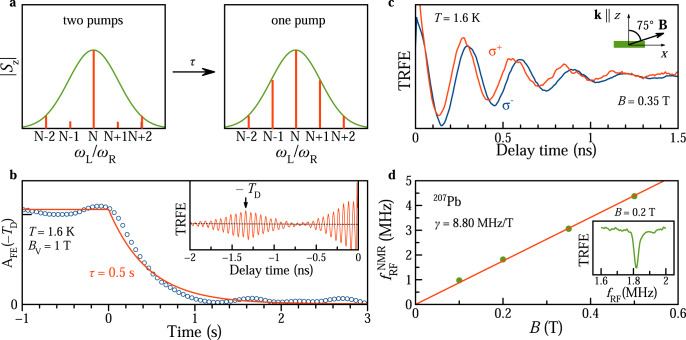
Hole-nuclei hyperfine interaction. **a** Schematic representation of the distribution ∣*S*_*z*_∣ of spin precession modes within the *g*-factor spread (green Gaussian) in the two-pump compared to the one-pump protocol. **b** Relaxation dynamics of the burst amplitude *A*_FE_(−*T*_D_) measured by switching from two-pump to one-pump protocol. *T* = 1.6 K and *B*_V_ = 1 T. Exponential fit gives decay time of the burst amplitude *τ* = 0.5 s, corresponding to the changeover time between the distributions, after blocking the second pump. Inset shows TRFE for negative time delays in the two-pump protocol. Arrow at delay of −*T*_D_ = −1.32 ns marks the position of the amplitude relaxation measurement. **c** TRFE signal for two circular polarizations of pump: *σ*^+^ (red) and *σ*^−^ (blue). Magnetic field *B* = 0.35 T is tilted from the light propagation direction by the angle *α* = 75°. **d**, Magnetic field dependence of ODNMR frequency (dots). Line is a linear fit with slope matching the gyromagnetic ratio *γ* = 8.80 MHz/T of ^207^Pb. Inset shows typical resonance curve at *B* = 0.2 T.

A typical TRFE signal, measured at *T* = 1.6 K in *B*_V_ = 1 T, is shown in the upper part of Fig. 1b. It shows oscillations with the Larmor frequency *ω*_L_ which is a linear function of *B*_V_: *ω*_L_ = ∣*g*∣*μ*_*B*_*B*_V_/${\hslash}$. This allows us to evaluate the *g*-factor of ∣*g*∣ = 1.20. *ω*_L_ has no offset at zero magnetic field (Fig. S4a), which is a strong argument in favor of the presence of resident carriers in the NCs rather than of carriers bound in an exciton^23^. In the latter case, an offset equal to the electron–hole exchange would be expected. General theoretical arguments and the experiments on dynamic nuclear polarization (Fig. 2c, see details below) indicate that the *g*-factor has a positive sign. From its value and the strong changes of *ω*_L_ in presence of polarized nuclei we conclude that the signal is dominated by resident holes with *g*_*h*_ = +1.20 (see Supplementary Notes 1 and 2). Note, that the stronger hole-nuclear interaction compared to the electron-nuclear one is specific for lead halide perovskite semiconductors^35^, due to their “inverted” band structure in comparison to semiconductors like GaAs or CdTe.

The TRFE amplitude decays within a nanosecond due to spin dephasing in the ensemble of NCs showing a dispersion of Larmor frequencies. The spin dephasing time at *B*_V_ = 1 T is ${T}_{2}^{ * }=0.26$ ns, and it increases up to 0.5 ns at low magnetic fields. There it becomes limited by the hole interaction with the nuclear spin fluctuations. In stronger fields, ${T}_{2}^{ * }$ decreases as 1/*B*_V_ due to the *g*_*h*_ dispersion Δ*g* = 0.03 (Fig. S4b). By using the spin inertia technique^39^ we measured the longitudinal spin relaxation time *T*_1_ = 7.6 *μ*s in zero longitudinal magnetic field, which gives an estimate of the upper limit of *T*_2_ in the studied NCs (Supplementary Note 4).

### Spin mode locking in nanocrystal ensembles

The most striking feature of the TRFE signal (blue) in Fig. 1b is the presence of an oscillating signal at negative time delays, before the pump arrival. Its amplitude has a maximum at small negative delays and decays with increasing negative delay with the same dephasing time as at positive delays. For such a signal to occur, optically-induced spin coherence should be present in the NCs for times exceeding the repetition period of the laser pulses *T*_R_ = 13.2 ns, and can therefore be obtained for spin coherence times *T*_2_ ≥ *T*_R_. The same effect was observed in ensembles of singly charged (In,Ga)As/GaAs QDs with both negative and positive charging^29,31,40^. It is caused by synchronization of the Larmor spin precession in the ensemble of NC carriers with the periodic laser pumping, termed the spin mode locking effect^41^, an effect similar to the resonant spin amplification^42^.

The SML effect results from the efficient accumulation of spin polarization from carrier spins which Larmor frequencies *ω*_L_ are commensurate with the laser repetition period *T*_R_. These spin precession modes fulfill the phase synchronization condition (PSC): *ω*_L_ = *Nω*_R_ = 2*πN*/*T*_R_, with *N* being an integer. If the single spin coherence time exceeds *T*_R_, the spin polarization of these modes accumulates. Modes fulfilling the PSC are shown schematically in Fig. 1c, assuming a Gaussian distribution of the Larmor frequencies centered around *N* = 3 for the weight of individual contributions, as shown Fig. 1d by the green line with the spread of Larmor frequencies Δ*ω*. Δ*ω* is contributed by the *g*-factor dispersion and by the nuclear spin fluctuations (Supplementary Note 4). Within this distribution, the five essential PSC modes (red needles) are located.

The signal resulting from the sum of the five PSC modes is shown at the bottom of Fig. 1c. The maxima of the PSC mode signal coincide with the pump pulse arrival times, in between the signal is reduced with a symmetry relative to the center between the pump pulses: the ensemble spin polarization dephases after a pump pulse, but then revives before the next pump. The pump pulse excites each time all modes within the Gaussian distribution, but only the PSC modes are amplified from pulse to pulse and contribute to the SML signal at negative delays. Remarkably, the spin coherence time *T*_2_ of the holes in individual NCs can be determined from the ratio of the signal amplitudes at negative and positive delays. This requires a modeling approach, that we will present in the following.

It was shown for (In,Ga)As QDs that the spectrum of PSC modes and the shape of TRFE signal can be tailored by using a two-pump protocol, where an additional second pump pulse is applied with a time delay *T*_D_ with respect to the first pump pulse. In this case, an additional synchronization condition is introduced, as the amplified PSC modes have to satisfy two commensurability conditions: *ω*_L_ = 2*πN*/*T*_R_ and *ω*_L_ = 2*πM*/*T*_D_, with *M* being an integer^29,31^. In the experiment this leads to the emergence of signal bursts at multiple times of *T*_D_. This behaviour is indeed also found in the studied perovskite NCs, as one can see from the red colored signal in Fig. 1b and its zoom in Fig. 1e, measured with *T*_D_ = 0.1 *T*_R_ = 1.32 ns. Two bursts at −1.32 ns and +2.64 ns are seen very prominently. A scheme of the PSC mode spectrum modification for the two-pump case is given in Fig. 2a.

### Nuclei-induced frequency focusing

In semiconductors, the spin dynamics of electrons and holes are strongly influenced by their interaction with the nuclear spin system in which they are embedded^43^. Polarized nuclei provide the Overhauser field *B*_N_, which shifts the carrier Larmor precession frequency to *ω*_L_ = ∣*g*∣*μ*_*B*_(*B*_V_ + *B*_N_)/${\hslash}$. Thereby, spin precession modes in Fig. 1d, that do not match the PSC, can be shifted in their frequency such that they satisfy the PSC. This effect was found experimentally in (In,Ga)As QDs and identified as nuclei-induced frequency focusing (NIFF) of the carrier spin coherence^30^. The NIFF mechanism represents a positive feedback loop, that provides the required nuclear polarization in each QD, so that a large dot fraction in the ensemble is pushed into PSC modes. Due to the very slow nuclear spin relaxation at cryogenic temperatures, the NIFF configuration can be kept for hours.

In an ideal, very efficient, system as is the case for (In,Ga)As QDs with strong carrier-nuclei interaction, NIFF results in identical amplitudes of the TRFE signal at negative and positive time delays, meaning that all precession frequencies are focused on the PSC modes. This is obviously not the case for the studied perovskite NCs, see Fig. 1b. Still, NIFF is present as can be evidenced by the relaxation dynamics of the bunch amplitude at −1.32 ns delay when the second pump is blocked^30^. In Fig. 2b we demonstrate such a measurement. We find the decay time of *τ* = 0.5 s, which is related to the repolarization of the nuclear spin system from the two-pump to the one-pump protocol, sketched in Fig. 2a. Without nuclear involvement the amplitude of the signal would decay within the carrier spin coherence time *T*_2_ after switching off the second pump, as was the case for hole spins in (In,Ga)As QDs^40^.

### Dynamic nuclear polarization and ODNMR

To further justify the involvement of nuclear spins, we apply the method of dynamic nuclear polarization (DNP) in a titled magnetic field^44^. The spin polarization of optically-oriented carriers is transferred via the hyperfine interaction to the nuclear spin system. The Overhauser field of the polarized nuclei **B**_N_ acts back on the carriers and shifts their Larmor precession frequency, the detailed scheme can be found in Supplementary Note 2 and ref. ^35^. The shift of the Larmor frequency allows one to measure the nuclear spin polarization.

One can see in Fig. 2c, that for *σ*^+^ pump the Larmor precession becomes faster compared to the *σ*^−^ case. The relative frequency shift is considerable, which is typical for the strong hole-nuclei spin interaction in perovskites. It allows us to confirm that the TRFE signal is related to holes and to evaluate the Overhauser field of *B*_N_ = 5.8 mT. The sign of the observed Larmor frequency change corresponds to a positive hole *g*-factor.

The variation of the TRFE signals by the DNP effect allows us to use it for optical detection of nuclear magnetic resonance (ODNMR)^35^. For that, the time delay is fixed and the TRFE amplitude is measured as a function of the radio frequency of additional radiation with *f*_RF_ = 0.1−10 MHz. The nuclear spin system is depolarized when the energy *hf*_RF_ matches to the Zeeman splitting of a nuclear isotope *μ*_N_*g*_N_*B*, i.e. for a NMR. Here, *μ*_N_ is the nuclear magneton and *g*_N_ is the nuclear *g*-factor (Supplementary Table 1). In the experiment it is detected as a resonant decrease of the TRFE amplitude, as shown in the inset of Fig. 2d at *B* = 0.2 T. From a linear fit of the resonance frequency dependence on the magnetic field ${f}_{{{{{{{{\rm{RF}}}}}}}}}^{{{{{{{{\rm{NMR}}}}}}}}}(B)$, we evaluate the gyromagnetic ratio *γ* = *μ*_N_*g*_N_/${\hslash}$ = 8.80 MHz/T, see Fig. 2d. One can conclude from the Supplementary Table 1 that among the nuclear isotopes present in the CsPb(Cl, Br)_3_ NCs only the ^207^Pb isotope matches this value. The dominant role of ^207^Pb on the carrier spin dynamics was identified earlier in FA_0.9_Cs_0.1_PbI_2.8_Br_0.2_ crystals and the theoretical analysis establishes it as common feature for lead halide perovskites^35^.

### Theory of spin mode locking in perovskite NCs

The theory of spin mode locking was previously developed for self-assembled quantum dots^45^ and successfully applied to explain the experimental results^40,41,46^. However, the discovery of SML in perovskite nanocrystals provides new theoretical challenges. First, the generation of spin coherence in perovskite structures with an inverted band structure occurs on the basis of other selection rules^43^. Circularly polarized light generates an exciton with an electron and a hole, both having spin 1/2, instead of an exciton with an electron (1/2) and a heavy hole (3/2). Second, perovskite colloidal nanocrystals in an ensemble are randomly oriented. For a complete theoretical description, the anisotropy of the *g*-factor for a single NC and the possibility of generating spin coherence through positively and negatively charged trions have to be considered. Taking all these facts into account requires a significant adjustment of the theoretical model.

The model, which details are given in Supplementary Notes 6–10, has two key parts: the first one considers the spin coherence generation and its dynamics in magnetic field for a single nanocrystal with arbitrary oriented crystallographic axes. The second part accounts for the inhomogeneity of the NC ensemble, namely the NCs random orientation and the *g*-factor spread.

Without loss of generality we consider resident holes, that can be created by photo-charging, as in the experiment the SML of holes is observed. The resident carrier spin polarization, providing the pump-probe signal, is generated by a mechanism involving trions. The oriented spin polarization components are tilted relative to the direction of the magnetic field, precess about it in time, and decay with the spin coherence time *T*_2_. For *T*_2_ ≥ *T*_R_ the spin polarization accumulates by pumping with a train of optical pulses. The SML is an ensemble effect resulting from a large number of oscillating signals with frequencies commensurable with *T*_R_, therefore, the model takes into account the spread of the *g*-factors (Supplementary Note 8).

**Fig. 3:**
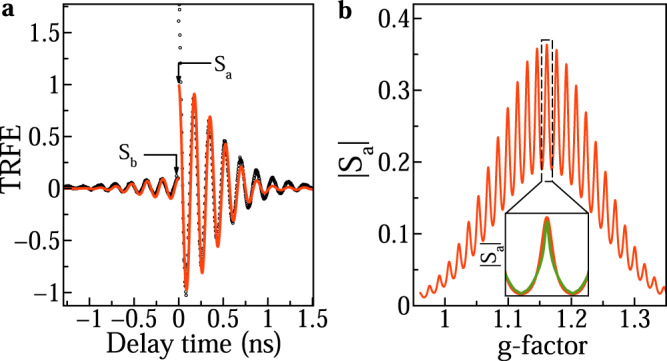
Calculation of spin polarization and TRFE signal. **a** Time-resolved Faraday ellipticity (dots) measured at *B*_V_ = 0.35 T and its modeling (red line). **b** Spectral distribution of precessing modes for *T*_2_ = 28 ns. Inset: illustration of precessing mode with NIFF for *T*_2_ = 13 ns (green) and without NIFF for *T*_2_ = 28 ns (red).

The experimentally observed spin dynamics, measured at *B*_V_ = 0.35 T (black dots in Fig. 3a), are modeled numerically using Supplementary Eqs. 28–61. The signal decay, at negative and positive time delays, is described by the ${T}_{2}^{ * }$, that is given by the *g*-factor spread, Δ*g*, and nuclear spin fluctuations, *δB*_N_. The ratio *S*_*b*_/*S*_*a*_ of the amplitudes before pulse arrival *S*_*b*_ and after pulse arrival *S*_*a*_ is determined by *T*_2_, the optical pulse area Θ and the NIFF effect. Technically, we calculate the spin polarization distribution *S*_*a*_ as a function of the Larmor frequency parameterized by the hole *g*-factor *ω*_L_ = *g*_*h*_*μ*_B_*B*_V_/${\hslash}$ (Fig. 3b). The distribution width is defined by 2Δ*g* with Δ*g* = 0.1. The multiple peaks correspond to synchronized precession modes, with the width of each peak determined by $1/{T}_{2}+1/{{{{{{{{\mathcal{T}}}}}}}}}_{2}^{ * }$, where ${{{{{{{{\mathcal{T}}}}}}}}}_{2}^{ * } \sim \hslash /({g}_{h}{\mu }_{{{{{{{{\rm{B}}}}}}}}}\sqrt{\delta {B}_{{{{{{{{\rm{N}}}}}}}}}^{2}})$ is related to the nuclear spin fluctuations. The modeled SML signal, corresponding to this distribution, is shown by the red line in Fig. 3a. It reproduces well all features of the experimental data. Finally we note, that the presented theoretical approach for the description of the SML effect is universal and applicable for different crystal phases of the NCs.

In order to highlight the role of the hole-nuclei interaction for the measured SML, we perform modeling without and with account for the NIFF. Without NIFF, we get a hole spin coherence time *T*_2_ = 28 ns for Θ = *π* as best fit parameter. The nuclear polarization changes the spin polarization distribution by increasing ${{{{{{{{\mathcal{T}}}}}}}}}_{2}^{ * }$ and narrowing the precession modes. This results in an increase of the ratio *S*_*b*_/*S*_*a*_, and to match its experimental value of 0.1 a shorter spin coherence time should be taken, to keep the mode width the same (insert of Fig. 3b). With NIFF we get *T*_2_ = 13 ns, which is at the lower limit of the times required for the SML to occur. It is remarkable that due to the involvement of NIFF we are able to observe the SML effect for such a short-lived hole spin coherence.

## Discussion

In summary, we have demonstrated the outstanding potential of perovskite NCs embedded in a glass matrix, using their spin properties as exemplary feature. We find a very long hole spin lifetime up to the microsecond range. We present an additional class of materials, in which the effects of spin mode locking and nuclei-induced frequency focusing are observed. This allows one to measure the hole spin coherence time *T*_2_, disregarding the random NC orientation and variation of NC sizes and shapes. It opens the pathway for implementation of multi-pulse protocols for manipulating the spin coherence, also with involvement of strong hole-nuclei interaction provided by the inverted band structure of the lead halide perovskites.

Despite our main goal here to report on the SML effect in perovskite NCs, we would like to compare our discovery with other systems. In the studies of colloidal CsPbBr_3_ NCs (grown in solution) the SML effect was not observed^22,23^. It demonstrates, that more investigations are needed to clarify whether it is related to some principal difference between colloidal NCs and NCs in glass, e.g. due to their very different surface conditions, and how this difference affects the spin coherence of carriers. However, the choice of a glass matrix or NC synthesis should not limit the observed effects fundamentally. We are convinced that they can be observed in different lead halide perovskite NCs, but also in lead-free NCs.

## Methods

### Samples

The studied CsPb(Cl, Br)_3_ nanocrystals embedded in a fluorophosphate ${{{{{{{\rm{Ba}}}}}}}}{({{{{{{{{\rm{PO}}}}}}}}}_{3})}_{2}$ glass matrix were synthesized by rapid cooling of a glass melt enriched with the components needed for the perovskite crystallization. Samples of fluorophosphates (FP) glasses of $60{{{{{{{\rm{Ba}}}}}}}}{({{{{{{{{\rm{PO}}}}}}}}}_{3})}_{2}-15{{{{{{{{\rm{NaPO}}}}}}}}}_{3}-12{{{{{{{{\rm{AlF}}}}}}}}}_{3}-1{{{{{{{{\rm{Ga}}}}}}}}}_{2}{{{{{{{{\rm{O}}}}}}}}}_{3}-4{{{{{{{{\rm{Cs}}}}}}}}}_{2}{{{{{{{\rm{O}}}}}}}}-8{{{{{{{{\rm{PbF}}}}}}}}}_{2}$ (mol. %) composition doped with 16 mol.% NaCl, 3.4 mol.% BaBr_2_ were prepared using the melt-quench technique. The glass synthesis was performed in a closed glassy carbon crucible at temperatures of 1000–1050 °C. About 50 g of the mixed powder was melted in the crucible during 20 min. Then the glass melt was cast on a glassy carbon plate and pressed to form a plate with a thickness of about 2 mm. The formation of the crystalline phase in glass can be carried out directly in the process of cooling the melt. During the pouring out of the transparent melt spontaneous precipitation of NCs with sizes smaller than the Bohr exciton radius occurs. By a subsequent heat treatment at a temperature just above the glass transition temperature (400–429 °C), it is possible to sequentially grow NCs to large sizes. The details of the method are given in ref. ^26^.

The NC size of 8 ± 1 nm is evaluated from a scanning transmission electron microscopy technique, see Supplementary Note 11. The concentration of NCs, of about 6 × 10^15^ cm^−3^, is evaluated from optical absorption measurements. The composition of the ${{{{{{{\rm{CsPb}}}}}}}}{({{{{{{{{\rm{Cl}}}}}}}}}_{0.56}{{{{{{{{\rm{Br}}}}}}}}}_{0.44})}_{3}$ NCs is estimated from the XRD data, see Supplementary Note 11. At *T* = 5 K the exciton resonance, mainly broadened due to the NC size dispersion, is at 2.743 eV. The band gap lies between that of bulk CsPbBr_3_ and bulk CsPbCl_3_. Additional for NCs, compared to the bulk case, the optical transition is shifted due to the quantum confinement^47,48^. For optical experiments, the sample with a thickness of 500 *μ*m was polished from both sides. The remarkable advantage of NCs in a glass matrix versus wet-chemistry synthesized NCs is the encapsulation resulting in long-term stability. Also the possibility to have optically flat sample surfaces with diminished light scattering is important for the polarization sensitive optical techniques used in this study. The effect of phase separation due to a laser excitation is excluded by low temperatures and relatively low laser powers applied resonantly to the NCs transitions. An additional long-term stability test is provided, in the Supplementary Note 11, to prove our claims.

### Magneto-optical measurements

For low-temperature optical measurements we use a liquid helium cryostat with a variable temperature from 1.6 K up to 300 K. At *T* = 1.6 K the sample is immersed in superfluid helium, while at 4.2–300 K it is held in helium gas. A superconducting vector magnet equipped with three orthogonal pairs of split coils to orient the magnetic field up to 3 T along any arbitrary direction is used. The magnetic field parallel to **k** is denoted as *B*_F_ (Faraday geometry), perpendicular to **k** as *B*_V_ (Voigt geometry). If not stated otherwise, the *B*_V_ is oriented horizontally. The angle *α* is defined as the angle between **B** and the light wave vector **k**, where *α* = 0° corresponds to **B**_F_.

### Transmission spectra

A white-light halogen lamp is used to measure the optical transmission. The sample was thinned to 43 *μ*m in order to avoid saturation of the light absorption in the spectral range of the exciton resonances. The spectra are detected by a 0.5 m monochromator equipped with a silicon charge-coupled-device camera.

### Time-resolved Faraday ellipticity (TRFE)

The coherent spin dynamics are measured using a degenerate pump-probe setup^31^. A titanium-sapphire (Ti:Sa) laser generates 1.5 ps long pulses in the spectral range of 700–980 nm (1.265–1.771 eV), which are frequency doubled with a beta barium borate crystal to the range of 350–490 nm (2.530–3.542 eV). The laser spectral width is about 1 nm (about 2 meV), and the pulse repetition rate is 76 MHz (repetition period *T*_R_ = 13.2 ns). The laser output is split into two beams, pump and probe, which pulses can be delayed with respect to one another by a mechanical delay line. The laser photon energy is tuned to be in resonance with the exciton transition, e.g. at 2.737 eV at *T* = 1.6 and 5 K. The pump and probe beams are modulated using photo-elastic modulators (PEM). The probe beam is linearly polarized and its amplitude is modulated at 84 kHz, while the pump beam is either helicity modulated at 50 kHz between *σ*^+^/*σ*^−^ circular polarizations or amplitude modulated at 100 kHz with the circular polarization fixed at either *σ*^+^ or *σ*^−^. The signal was measured by a lock-in amplifier locked to the difference frequency of the pump and probe modulation. For measuring by spin inertia technique, the modulation frequency of the pump helicity *f*_*m*_ was varied from 1 kHz to 5 MHz by an electro-optical modulator (EOM). For measuring the spin dynamics induced by the pump pulses, the polarization of the transmitted probe beam was analyzed with respect to its ellipticity (Faraday ellipticity) using balanced photodiodes and a lock-in amplifier. In *B*_V_ ≠ 0, the Faraday ellipticity amplitude oscillates in time reflecting the Larmor precession of the charge carrier spins. The dynamics of the Faraday ellipticity signal can be described by a decaying oscillatory function:

1 $${A}_{{{{{{{{\rm{FE}}}}}}}}}(t)=S\cos ({\omega }_{{{{{{{{\rm{L}}}}}}}}}t)\exp (-t/{T}_{2}^{ * }).$$

Here *S* is the amplitude at zero delay, *ω*_L_ is the Larmor precession frequency, and ${T}_{2}^{ * }$ the spin dephasing time. For application of a two-pump pulse scheme, after the delay line the so far not modulated linearly polarized pump beam was passed through a 50/50 nonpolarizing beam splitter. The deflected beam was sent via two mirrors over a certain distance and merged with the transmitted pump beam via a second nonpolarizing 50/50 beam splitter. By careful adjustment both pump beams were brought aligned parallel to each other, to excite the same sample spot with 300 *μ*m diameter.

### Pump-probe time-resolved differential transmission

Here, for excitation by the pump beam, the same scheme as in TRFE is used, but with linear polarization. The pump is amplitude modulated by a photo-elastic modulator at 100 kHz. The linearly polarized, unmodulated probe beam is separated into a reference beam, which is routed around the cryostat and the test beam, which is transmitted through the sample. The signal is recorded with respect to the intensity difference between the transmitted and the reference beams, using a balanced photodetector.

### Optically-detected nuclear magnetic resonance (ODNMR) with TRFE detection

In this technique nuclear magnetic resonances are measured via resonant decrease of dynamic nuclear polarization by radio frequency (RF) radiation. For that optical detection of the TRFE amplitude at fixed magnetic field is used. A small RF coil of about 5 mm diameter having 5 turns is placed close to the sample surface, similar to ref. ^49^. The coil is mounted flat at the sample surface and the laser beam is transmitted through the bore of the coil. The RF induced magnetic field direction is perpendicular to its surface and parallel to **k**. The RF in the frequency range *f*_RF_ from 100 Hz up to 10 MHz is driven by a frequency generator, with an applied voltage of 10 V leading to an oscillating field amplitude of about 0.1 mT. The RF is terminated by internal 50Ω resistors, but not frequency matched to the circuit. In the low frequency range up to 5 MHz the current is nearly frequency independent, as the inductive resistance of the coil is small compared to the internal termination. For observation of DNP it is essential to tilt the external magnetic field away from the Voigt geometry, to have a nonzero scalar product **B** ⋅ **S** and apply a constant pump helicity.

## Supplementary information

Supplementary Information

## Data Availability

The data on which the plots in this paper are based and other findings of this study are available from the corresponding authors upon justified request.

## Code availability

The code on which the calculations within this paper are based and other findings of this study are available from the corresponding author upon justified request.

## References

[1] Jena AK, Kulkarni A, Miyasaka T (2019). Halide perovskite photovoltaics: background, status, and future prospects. Chem. Rev..

[2] Best Research - Cell Efficiency Chart, https://www.nrel.gov/pv/cell-efficiency.html (2021).

[3] *Halide Perovskites for Photonic.* (eds. Vinattieri, A. & Giorgi, G.) (AIP Publishing, Melville, New York, 2021).

[4] *Hybrid Organic Inorganic Perovskites: Physical Properties and Applications.* (eds. Vardeny, Z. V. & Beard, M. C.) (World Scientific, 2022).

[5] Wang J (2019). Spin-optoelectronic devices based on hybrid organic-inorganic trihalide perovskites. Nat. Commun..

[6] Ning W (2020). Magnetizing lead free halide double perovskites. Sci. Adv..

[7] Kim Y-H (2021). Chiral-induced spin selectivity enables a room-temperature spin light-emitting diode. Science.

[8] Kovalenko MV, Protesescu L, Bondarchuk MI (2017). Properties and potential optoelectronic applications of lead halide perovskite nanocrystals. Science.

[9] Chen Q (2018). All-inorganic perovskite nanocrystal scintillators. Nature.

[10] Protesescu L (2015). Nanocrystals of cesium lead halide perovskites (CsPbX_3_, X = Cl, Br, and I): novel optoelectronic materials showing bright emission with wide color gamut. Nano Lett..

[11] Akkerman QA, Rainó G, Kovalenko MV, Manna L (2018). Genesis, challenges and opportunities for colloidal lead halide perovskite nanocrystals. Nat. Mater..

[12] Park Y-S, Guo S, Makarov NS, Klimov VI (2015). Room temperature single-photon emission from individual perovskite quantum dots. ACS Nano.

[13] Yu B (2021). Ultrafast dynamics of photoexcited carriers in perovskite semiconductor nanocrystals. Nanophotonics.

[14] Becker MA (2018). Bright triplet excitons in caesium lead halide perovskites. Nature.

[15] Lin J (2016). Direct observation of band structure modifications in nanocrystals of CsPbBr_3_ perovskite. Nano Lett..

[16] Tamarat P (2019). The ground exciton state of formamidinium lead bromide perovskite nanocrystals is a singlet dark state. Nat. Mater..

[17] Ramade J (2018). Fine structure of excitons and electron-hole exchange energy in polymorphic CsPbBr_3_ single nanocrystals. Nanoscale.

[18] Fu M (2017). Neutral and charged exciton fine structure in single lead halide perovskite nanocrystals revealed by magneto-optical spectroscopy. Nano Lett..

[19] Isarov M (2017). Rashba effect in a single colloidal CsPbBr_3_ perovskite nanocrystal detected by magneto-optical measurements. Nano Lett..

[20] Canneson D (2017). Negatively charged and dark excitons in CsPbBr_3_ perovskite nanocrystals revealed by high magnetic fields. Nano Lett..

[21] Nestoklon MO (2018). Optical orientation and alignment of excitons in ensembles of inorganic perovskite nanocrystals. Phys. Rev. B.

[22] Crane MJ (2020). Coherent spin precession and lifetime-limited spin dephasing in CsPbBr_3_ perovskite nanocrystals. Nano Lett..

[23] Grigoryev PS, Belykh VV, Yakovlev DR, Lhuillier E, Bayer M (2021). Coherent spin dynamics of electrons and holes in CsPbBr_3_ colloidal nanocrystals. Nano Lett..

[24] Strohmair S (2020). Spin polarization dynamics of free charge carriers in CsPbI_3_ nanocrystals. Nano Lett..

[25] Chen D, Yuan S, Chen J, Zhong J, Xu X (2018). Robust CsPbX_3_ (X = Cl, Br, and I) perovskite quantum dot embedded glasses. J. Mater. Chem. C..

[26] Kolobkova EV, Kuznetsova MS, Nikonorov NV (2021). Perovskite CsPbX_3_ (X = Cl, Br, I) nanocrystals in fluorophosphate glasses. J. Non-Cryst. Solids.

[27] Press D, Ladd TD, Zhang B, Yamamoto Y (2008). Complete quantum control of a single quantum dot spin using ultrafast optical pulses. Nature.

[28] Greilich A (2009). Ultrafast optical rotations of electron spins in quantum dots. Nat. Phys..

[29] Greilich A (2006). Mode locking of electron spin coherences in singly charged quantum dots. Science.

[30] Greilich A (2007). Nuclei-induced frequency focusing of electron spin coherence. Science.

[31] Yakovlev, D. R. & Bayer, M. Coherent spin dynamics of carriers, Chapter 6 in *Spin Physics in Semiconductors* (ed. Dyakonov, M. I.) pp. 155–206 (Springer International Publishing AG, 2017).

[32] Evers E (2021). Suppression of nuclear spin fluctuations in an InGaAs quantum dot ensemble by GHz-pulsed optical excitation. npj Quantum Inf..

[33] Evers E (2021). Shielding of external magnetic field by dynamic nuclear polarization in (In,Ga)As quantum dots. Phys. Rev. B.

[34] Belykh VV (2019). Coherent spin dynamics of electrons and holes in CsPbBr_3_ perovskite crystals. Nat. Commun..

[35] Kirstein E (2022). Lead-dominated hyperfine interaction impacting the carrier spin dynamics in halide perovskites. Adv. Mater..

[36] Kirstein, E. et al. The Landé factors of electrons and holes in lead halide perovskites: universal dependence on the band gap. *Nat. Commun.* **13**, 3062 (2022). 10.1038/s41467-022-30701-0 PMC9163162 35654813

[37] Odenthal P (2017). Spin-polarized exciton quantum beating in hybrid organic–inorganic perovskites. Nat. Phys..

[38] Garcia-Arellano G (2021). Energy tuning of electronic spin coherent evolution in methylammonium lead iodide perovskites. J. Phys. Chem. Lett..

[39] Smirnov DS (2020). Spin polarization recovery and Hanle effect for charge carriers interacting with nuclear spins in semiconductors. Phys. Rev. B.

[40] Varwig S (2012). Hole spin precession in a (In,Ga)As quantum dot ensemble: from resonant spin amplification to spin mode locking. Phys. Rev. B.

[41] Yugova IA, Glazov MM, Yakovlev DR, Sokolova AA, Bayer M (2012). Coherent spin dynamics of electrons and holes in semiconductor quantum wells and quantum dots under periodical optical excitation: resonant spin amplification versus spin mode locking. Phys. Rev. B.

[42] Kikkawa JM, Awschalom DD (1998). Resonant spin amplification in n-type GaAs. Phys. Rev. Lett..

[43] Glazov, M. M. *Electron & Nuclear Spin Dynamics in Semiconductor Nanostructures* (Oxford University Press, Oxford, UK, 2018).

[44] Kalevich VK, Korenev VL (1991). Optical polarization of nuclei and ODNMR in GaAs/AlGaAs quantum wells. Appl. Magn. Reson..

[45] Yugova IA, Glazov MM, Ivchenko EL, Efros AlL (2009). Pump-probe Faraday rotation and ellipticity in an ensemble of singly charged quantum dots. Phys. Rev. B..

[46] Fras F (2012). Hole spin mode locking and coherent dynamics in a largely inhomogeneous ensemble of *p*-doped InAs quantum dots. Phys. Rev. B.

[47] Krieg F (2021). Monodisperse long-chain sulfobetaine-capped CsPbBr_3_ nanocrystals and their superfluorescent assemblies. ACS Cent. Sci..

[48] Dong Y (2018). Precise control of quantum confinement in cesium lead halide perovskite quantum dots via thermodynamic equilibrium. Nano Lett..

[49] Heisterkamp F (2016). Dynamics of nuclear spin polarization induced and detected by coherently precessing electron spins in fluorine-doped ZnSe. Phys. Rev. B.

