# Supplementary material for: Mode locking of hole spin coherences in CsPb(Cl, Br)3 perovskite nanocrystals
Source: Nat Commun. 2023 Feb 8;14:699. doi: 10.1038/s41467-023-36165-0 (PMC9908866; doi:10.1038/s41467-023-36165-0)
Supplement: Supplementary file 1 — Supplementary Information [file 41467_2023_36165_MOESM1_ESM.pdf]

Supplementary Information:
Mode locking of hole spin coherences in $CsPb(Cl,Br)_3$ perovskite nanocrystals

E. Kirstein, N. E. Kopteva, D. R. Yakovlev, E. A. Zhukov, E. V. Kolobkova,
M. S. Kuznetsova, V. V. Belykh, I. A. Yugova, M. M. Glazov, M. Bayer, and A. Greilich

SUPPLEMENTARY NOTE 1: HOLE g-FACTOR IN NANOCRYSTALS IN COMPARISON WITH UNIVERSAL DEPENDENCE ON BAND GAP FOR BULK PEROVSKITES

For a convenient comparison of the g-factors in NCs, with bulk perovskites, we use for the NCs the same approach for the definition of the g-factor sign as in the bulk, see e.g. Refs. [1–3]. In this definition the g-factors are positive both for electrons and holes when their ground state is the state with a spin projection $S_z = -1/2$ onto the direction of the magnetic field. In this approach the bright exciton g-factor equals $g_X = g_e + g_h$. We used the same approach in our recent paper on electron and hole spin coherence in $CsPbBr_3$ NCs [4], while in our earlier paper [5] on the polarized photoluminescence in strong magnetic fields from $CsPbBr_3$ NCs another approach, common for colloidal NCs [6], was taken, which gives the opposite sign for the hole g-factor. Supplementary Figure 1 shows the experimentally measured values of g-factors for electrons and holes in perovskite crystals versus the corresponding band gap energies (E_g). The hole g-factor value $g_h = +1.20$ in $CsPb(Cl,Br)_3$ NCs from the present study coincides well with the expected behavior.

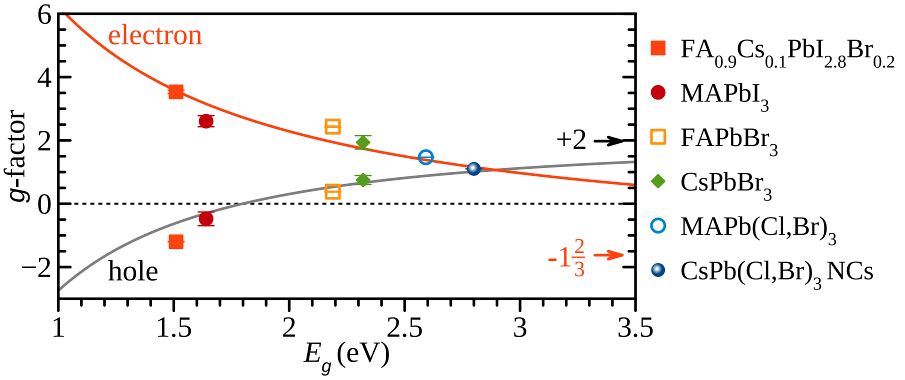

Supplementary Figure 1. **Electron and hole g-factors versus band gap energy in lead halide perovskite crystals.** Experimental data for various bulk perovskites measured at $T = 1.6$ and 5 K are taken from Ref. [7] and shown by the symbols with error bars. Solid lines are model calculations, for details see Ref. [7]. The limiting values of the electron ($-5/3$) and hole ($+2$) g-factors for $E_g \to \infty$ are given by the arrows. Blue circle shows the hole g-factor in the $CsPb(Cl_{0.56}Br_{0.44})_3$ NCs with $g_h = +1.20$, measured in the present study.

SUPPLEMENTARY NOTE 2: DYNAMIC NUCLEAR POLARIZATION IN PEROVSKITES WITH $g_e > 0$ AND $g_h > 0$

Let us consider a dynamic nuclear polarization by spin-oriented holes. It can not be realized in the Voigt geometry, as one need to have a finite projection of the hole spin polarization on the direction of the external magnetic field **B** (Fig. 2). The circularly polarized pump pulses orient the hole spin (i.e. generate the hole spin polarization) along the direction of the k-vector of light (i.e. along the optical axis), see the green arrows in Fig. 2. The hole spin polarization **S** is transferred by a flip-flop processes to the nuclear spin system. The nuclear polarization **I** is collinear with the external magnetic field **B** and its orientation is controlled by the orientation of **S**, i.e. can be controlled by the pump helicity. The oriented nuclear spins (**I**) create an effective magnetic field acting back on the hole spins – the Overhauser field ($\mathbf{B}_\mathrm{N}$) [8]. If the hole spin is in a tilted external magnetic field (**B**), the Overhauser field has a nonzero projection onto the direction of the external field and consequently increases or decreases the Larmor frequency of the hole spin precession. This depends on the pump circular polarization and therefore on the initial hole spin orientation, see Fig. 2.

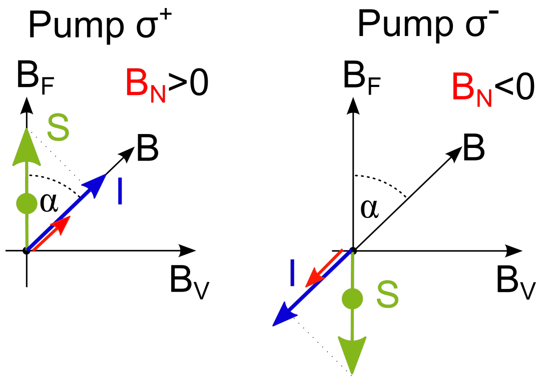

Supplementary Figure 2. **Dynamic nuclear polarization in perovskites with $g_e > 0$ and $g_h > 0$ in tilted magnetic field.** Scheme of interaction of charge carrier spins with nuclear spin system (identical for electrons and holes). Optically polarized carriers transfer their spin to the nuclei and induce nuclear spin polarization **I**. In turn, the nuclear polarization causes an Overhauser field, $\mathbf{B}_\mathrm{N}$, acting back on the carriers. Orientation of the carrier spin polarization **S** (green arrow) is determined by the light helicity, σ^+ (σ^-) polarizations causes spin up (down), shown in the left and right panels, respectively. The nuclear polarization **I** (blue arrow) builds up in the direction of **S**, along the magnetic field **B** (black arrow). Here the magnetic field is inclined relative to the carrier spin polarization by an angle α. For a σ^+ polarized pump, the nuclear spin polarization manifests as Overhauser field $\mathbf{B}_\mathrm{N}$ (red arrow), which is directed along **I** and along **B**. For a σ^- polarized pump $\mathbf{B}_\mathrm{N}$ and **B** are antiparallel to each other.

Supplementary Table 1. Major abundant non-zero nuclear spin isotopes in $CsPb(Cl,Br)_3$. The table columns give: isotope name, natural abundance α, nuclear spin I, magnetic dipole moment of the isotope μ normalized to the nuclear magneton μ_N, gyromagnetic ratio γ. Note that $\mu = g_\mathrm{N}\mu_\mathrm{N}$ and $\gamma = g_\mathrm{N}\mu_\mathrm{N}/\hbar$, where g_N is nuclear g-factor.

isotope	α	I	μ/μ_N	γ [MHz/T]
^{133}Cs	100%	7/2	2.58	5.623
^{207}Pb	22.1%	1/2	0.58	8.882
^{35}Cl	75.8%	3/2	0.82	4.176
^{37}Cl	24.2%	3/2	0.68	3.476
^{79}Br	50.7%	3/2	2.10	10.704
^{81}Br	49.3%	3/2	2.27	11.538

Note, that for $CsPb(Cl,Br)_3$ NCs, where the electron and hole g-factors are positive ($g_e > 0$ and $g_h > 0$), the schemes shown in Fig. 2 are the same for the electron and hole. A change of the g-factor sign inverts the direction of the carrier spin polarization **S** and, respectively, the direction of the nuclear polarization **I**.

Which nuclear spins are affected by the carrier spins, depends on various parameters [3]. The most important ones for the abundant non-zero nuclear spin isotopes in $CsPb(Cl,Br)_3$ are given in Tab. 1.

SUPPLEMENTARY NOTE 3: TEMPERATURE DEPENDENCE OF HOLE SPIN MODE LOCKING

As our previous studies show [1], the efficiency of the dynamic nuclear polarization strongly depends on the temperature and the excitation power, which should also be seen via a decreased efficiency of the NIFF. In the case of the studied perovskite NCs, a temperature increase from $T = 1.6$ K to 5.3 K results in a loss of the SML signal amplitude at negative delay times in the TRFE signal (Fig. 3). Taking into account the strong hole localization and constant pump power, we associate this effect with a reduction of the NIFF effect and/or a shortening of the hole spin coherence time T_2. This leads to a decrease of the hole SML amplitude for $T_2 \leq T_\mathrm{R}$, as it was observed in (In,Ga)As/GaAs quantum dots [10, 11]. The main reason for a fast coherence decay with an elevated temperature, can be related to the spectral diffusion of the nuclear spin distribution due to the excitation of acoustic phonons [10].

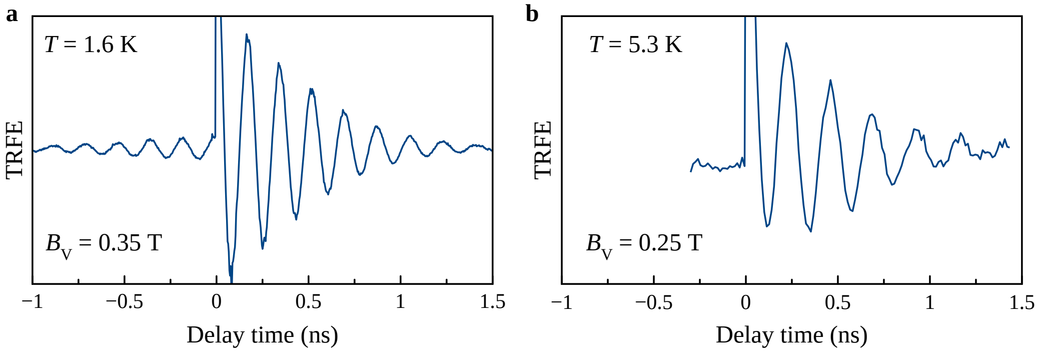

Supplementary Figure 3. **Temperature dependence of hole spin mode locking.** Time-resolved Faraday ellipticity signal measured at $E_{\rm pu} = 2.737\,$eV in the one-pump protocol. **a**, $B_{\rm V} = 0.35\,$T and $T = 1.6\,$K. **b**, $B_{\rm V} = 0.25\,$T and $T = 5.3\,$K. Pump helicity was modulated at a frequency $f_m = 10\,$kHz.

SUPPLEMENTARY NOTE 4: SPIN DEPHASING AND SPIN RELAXATION OF HOLES IN $CsPb(Cl,Br)_3$ NANOCRYSTALS

Supplementary Figure 3 shows the TRFE signal, revealing a single-frequency damped oscillation at $T = 5.3\,$K and $B_{\rm V} = 0.25\,$T. The magnetic field dependence of the Larmor precession frequency $\omega_{\rm L}$ is shown in Fig. 4**a**. The dependence of $\omega_{\rm L}(B_{\rm V})$ is linear without any offset at zero field, $\omega_{\rm L} = |g_h|\mu_B B_{\rm V}/\hbar$. It allows us to determine the hole g-factor value of $|g_h| = 1.20$ in the $CsPb(Cl_{0.56}Br_{0.44})_3$ NCs. The right axis in Fig. 4**a** shows the value of the hole Zeeman splitting $E_Z = \hbar\omega_{\rm L}$. According to the theoretical dependence of the g-factor on the band gap, given in Supplementary Note 1, we assign a positive sign to the hole g-factor. The hole spin dephasing time T_2^* shortens with an increase of $B_{\rm V}$ due to an increased precession phase mismatch caused by the g-factor spread Δg (Fig. 4b). This dependence can be described by the equation:

$$T_2^* = \frac{\hbar}{\sqrt{(\Delta g \mu_B B_{\rm V})^2 + (g_h \mu_B \Delta B)^2}}, \quad (1)$$

from which the spread of g-factors $\Delta g = 0.03$ and the spin dephasing time $T_2^*(B_{\rm V} = 0) = \hbar/(g_h \mu_B \Delta B) = 0.5\,$ns due to the hole spin relaxation in zero external magnetic field by the nuclear spin fluctuation field $\Delta B = 20\,$mT are determined. Supplementary Figure 4c presents the spin dephasing rate $1/T_2^*$ as function of the applied magnetic field.

In zero magnetic field, the hole spin polarization relaxes due to interaction with random nuclear spin fluctuations.

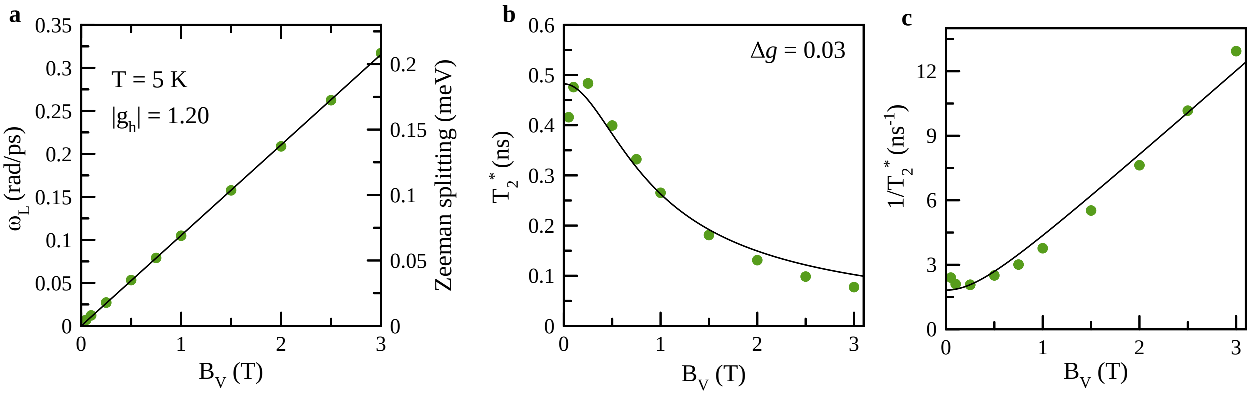

Supplementary Figure 4. **Magnetic field dependencies.** **a**, Magnetic field dependence of the Larmor precession frequency measured at $T = 5\,$K for pump photon energy of $2.737\,$eV (green circles), $f_m = 10\,$kHz. Linear fit (solid line) gives $|g_h| = 1.20$. **b**, Spin dephasing time T_2^* and **c**, spin dephasing rate $1/T_2^*$, as functions of the magnetic field measured at $T = 5\,$K. Lines are fits with Eq. (1), using the parameters $T_2^*(B_{\rm V} = 0) = 0.5\,$ns and $\Delta g = 0.03$.

However, application of an external longitudinal magnetic field along the optical axis leads to a restoration of the spin polarization as shown in Fig. 5a by the polarization recovery curve (PRC).

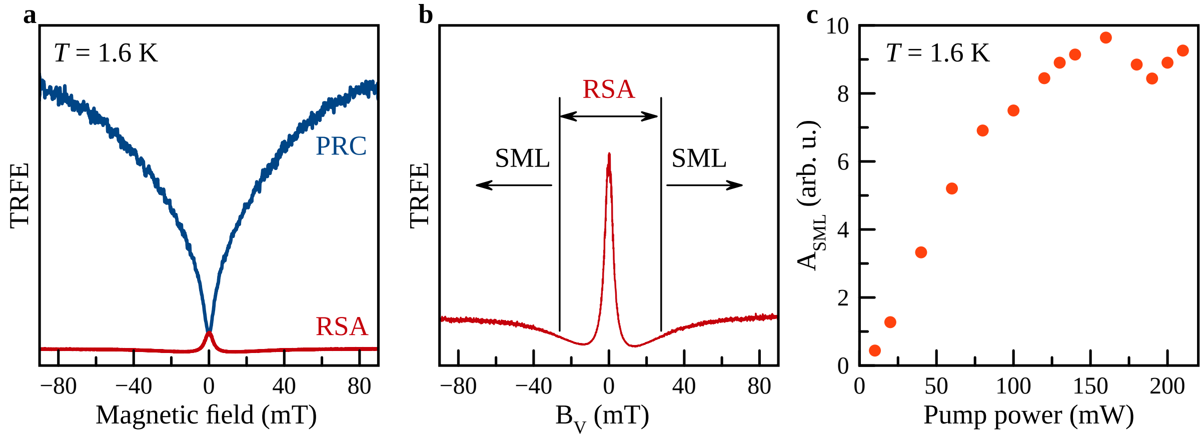

Supplementary Figure 5. **PRC and RSA dependencies.** **a**, PRC (blue) measured in B_{F}, and RSA (red) measured in B_{V} at the negative probe delay $t = -10$ ps for $T = 1.6$ K. Laser photon energy is 2.737 eV, pump power is 20 mW at the modulation frequency of $f_m = 10$ kHz. **b**, Zoom of the RSA signal from panel **a**. With increasing magnetic field the RSA regime is changing over to the SML regime. **c**, Spin mode locking amplitude A_{SML} at $t < 0$ as a function of the pump power measured in the one-pump protocol with $T_R = 13.2$ ns.

The spin polarization can be decreased by a transverse magnetic field. However, due to the pulsed excitation, when the spin of a resident carrier undergoes an integer number of revolutions about the external magnetic field between subsequent pump pulse arrivals, the magnitude of spin polarization measured close to zero delay increases again - this is the case of resonant spin amplification (RSA) [9, 24, 25]. If the spin ensemble has a significant spread of g-factors, the RSA effect occurs only in a narrow range of magnetic fields around zero. Further increase of the magnetic field leads to an increasing number of possible PSC precessing modes, and the polarization magnitude becomes nonzero, reaching a constant value - the SML effect, as shown in Fig. 5a and its zoom in Fig. 5b. Such a transition of the hole spin system from RSA to SML was reported before for (In,Ga)As quantum dots with p-type doping [12].

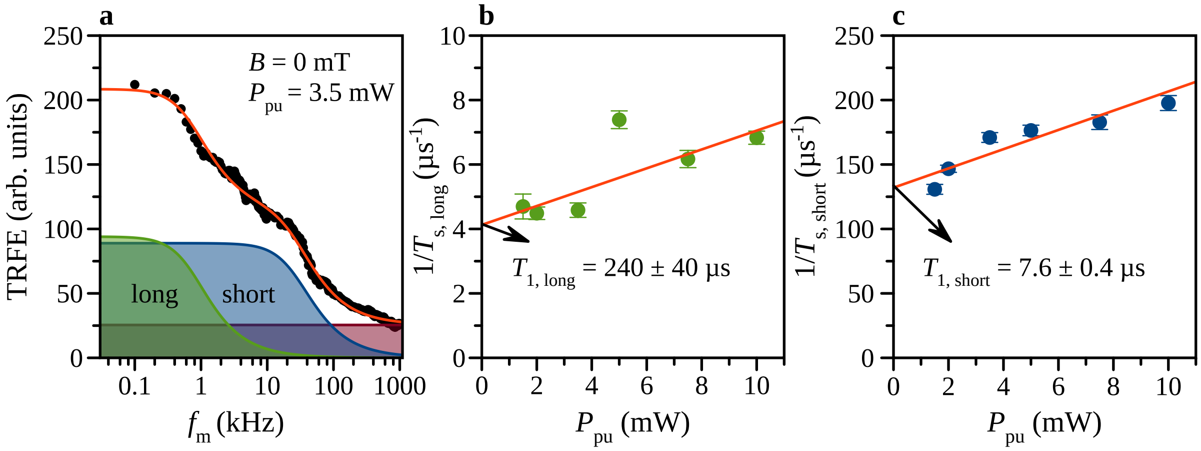

Supplementary Figure 6. **Spin inertia.** **a**, Faraday ellipticity amplitude as a function of the pump modulation frequency f_{m} for a pump power of $P_{\mathrm{pu}} = 3.5$ mW measured at a magnetic field of $B = 0$ mT with a pump-probe delay of -10 ps (black circles). The red line is the two-component fit. The contributions of each component are shown by the green-shaded and blue-shaded areas. The violet-shaded area gives the frequency independent offset, that can be related to the scattered light. **b**–**c**, The power dependence of the two corresponding inverse effective spin lifetimes $1/T_s$. A linear extrapolation to zero power (red lines) yields $T_{1,\mathrm{long}} = (240 \pm 40)\,\mu$s (**b**) and $T_{1,\mathrm{short}} = (7.6 \pm 0.4)\,\mu$s (**c**). $T = 1.6$ K. All error bars are given by the standard deviations from the fits.

The SML effect strongly depends on the pump power, as shown in Fig. 5c. The SML amplitude increases and reaches saturation as the pump power increases. We did not observe in perovskite NCs Rabi oscillations, which have been reported for singly-charged (In,Ga)As quantum dots [13]. Their absence is related to the pronounced inhomogeneity of the studied system.

To complete the characterization of the spin dynamics we have measured the longitudinal spin relaxation time T_1, using the spin inertia technique, see Fig. 6 [14, 15]. Here, we alternate the pump helicity between σ^+ and σ^- and measure the spin polarization response with respect to the frequency of modulation, f_m. By increasing the frequency one can enter a regime where the modulation period is shorter than the spin relaxation time T_1. Thus the signal amplitude drops [14]. The decay of the ellipticity signal upon increasing f_m can be described by the dependence $S(f_\mathrm{m}) = S_0/\sqrt{1+(2\pi f_\mathrm{m} T_s)^2}$, where T_s is the effective spin lifetime at the corresponding pump power. The extrapolation of T_s to zero power allows for extracting the intrinsic spin relaxation T_1 of the carriers. Supplementary Figures 6(b) and 6(c) depict the extracted times for two components present in the spin-inertia dependence. We relate them to two subsets of carriers in the NC ensemble and focus on the regime $f_\mathrm{m} \geq 10\,\mathrm{kHz}$ for which only the shorter living component with $T_1 = 7.6\,\mu\mathrm{s}$ contributes significantly.

SUPPLEMENTARY NOTE 5: THEORY OF SPIN MODE LOCKING. INTRODUCTION

The theory of spin mode locking was previously developed for singly charged epitaxial quantum dots [9] and successfully applied to model experimental data in (In,Ga)As QDs [12, 13]. However, discovering spin-locked modes in perovskite nanocrystals has provided new theoretical challenges. First, the generation of spin coherence in perovskite structures with an inverted band structure occurs on the basis of modified selection rules. Circularly polarized light generates excitons with an electron ($S = 1/2$) and a hole ($S = 1/2$), instead of an exciton with an electron ($S = 1/2$) and a heavy hole ($S = 3/2$). Second, an ensemble of perovskite colloidal nanocrystals has a random orientation of the c-axis, which leads to less efficient generation of hole spin polarization along the optical axis. The anisotropy c-axis is typical for the low temperatures when the perovskite materials expose structural transition from cubic to tetragonal or orthorhombic phase. Third, due to the variety of perovskite materials, different nanocrystal samples may have different crystal symmetries, which is also considered in the developed model, even though the calculations are performed here only for cubic symmetry. For a complete theoretical description, the anisotropy of the g-factor for a single NC has to be considered, as well as the possibility of generating spin coherence through positively and negatively charged trions. In order to account for all these factors a considerable modification of the previously developed models of SML and NIFF need to be done.

The theoretical part is structured as follows:

1) Section Supplementary Note 5 introduces the choice of the used coordinate systems.

2) In section Supplementary Note 6 the generation of spin polarization of resident carriers (electron and hole) through an intermediate trion state in a single NC is considered.

3) Section Supplementary Note 7 considers the universal electron and hole spin polarization behavior in an external

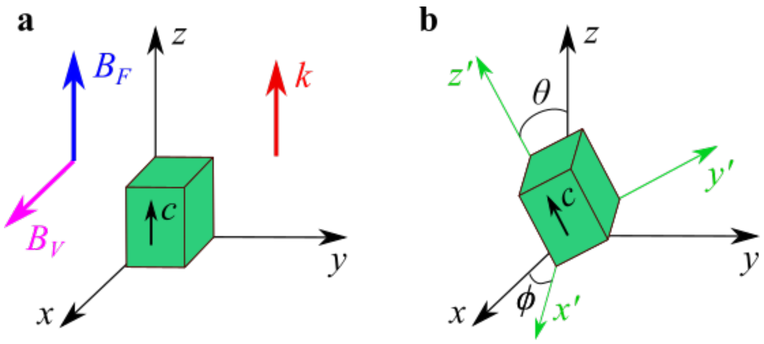

Supplementary Figure 7. **Laboratory and NC coordinate systems used for the calculations.** **a**, Laboratory coordinate system (x, y, z) where $\mathbf{k} \parallel z$, $\mathbf{B}_\mathrm{F} \parallel z$ and $\mathbf{B}_\mathrm{V} \parallel x$ for the Faraday and Voigt geometries, respectively. The green parallelepiped symbolizes a single nanocrystal with the c-axis oriented parallel to the k-vector ($c \parallel \mathbf{k}$). **b**, Illustration of the NC coordinate frame (x', y', z') tilted with respect to (x, y, z). Orientation angles $\sphericalangle(z, z') := \theta \in [0; \pi]$ and $\sphericalangle(x, x') := \phi \in [0; 2\pi]$ are defined as shown in the scheme.

magnetic field for a single NC with an anisotropic g-factor.

4) Section Supplementary Note 8 considers the inhomogeneity of the NC ensemble due to the g-factor dispersion and random orientation of the NC axes.

5) The modeling of the experimental results is given in Sections Supplementary Note 9 and Supplementary Note 10.

Let us start the presentation of the theoretical model by introducing the coordinate systems. The laboratory coordinate system is taken as (x, y, z) where $\mathbf{k} \parallel z$, $\mathbf{B}_\text{F} \parallel z$ and $\mathbf{B}_\text{V} \parallel x$ for the Faraday and Voigt geometries, shown in Fig. 7a. The single nanocrystal with $c \parallel z$ is presented by the green parallelepiped. For this NC orientation, the Voigt geometry is $\mathbf{B}_\text{V} \parallel x$ and $\mathbf{k} \parallel z$. The coordinate system (x', y', z') is associated with the NC axes and $c \parallel z'$ as shown in Fig. 7b. (x', y', z') is tilted in respect to (x, y, z) by the angles $\theta \in [0; \pi]$ and $\phi \in [0; 2\pi]$, for a NC with an arbitrary c-axis orientation. The final expressions for the spin polarization components are integrated over all possible projections of the external magnetic field and the light propagation direction on the NC axes, to simulate the random orientation of the NC axes relative to the laboratory coordinate system. One can project (x, y, z) on (x', y', z'):

$$x' = (x \cos\theta - z \sin\theta) \cos\phi + y \sin\phi, \tag{2}$$

$$y' = -(x \cos\theta - z \sin\theta) \sin\phi + y \cos\phi, \tag{3}$$

$$z' = z \cos\theta + x \sin\theta. \tag{4}$$

We assume that the laser light propagates along z ($\mathbf{k} \parallel z$), so that its electric field component $E_z = 0$. Therefore, the electric field components in the NC coordinate system (x', y', z') are:

$$E_{x'} = E_x \cos\theta \cos\phi + E_y \sin\phi, \tag{5}$$

$$E_{y'} = -E_x \cos\theta \sin\phi + E_y \cos\phi, \tag{6}$$

$$E_{z'} = E_x \sin\theta. \tag{7}$$

For the σ^+ circularly polarized light $E_x = iE_y$.

SUPPLEMENTARY NOTE 6: OPTICAL GENERATION OF SPIN COHERENCE

This section considers the generation of spin polarization of resident carriers (electrons and holes) in a single NC via intermediate trion states using short optical pulses. Atomistic modeling [17] shows that the s-orbitals of the metal (Pb) form the valence band with an admixture of the p-orbitals of the halogen (Br,Cl). The p-orbitals of the metal form the conduction band with an admixture of the halogen s-orbitals [1]. Taking into account the spin-orbit interaction, the electron $\pm 1/2$ spin states in the conduction band and the hole $\pm 1/2$ spin states in the valence band determine the fundamental optical transitions [1], see also Fig. 8.

The matrix elements of the momentum operator $\hat{p} = (p_x, p_y, p_z)$ taken between the conduction band Bloch functions $|c.b., x\rangle$, $|c.b., y\rangle$ or $|c.b., z\rangle$ and the valence band $|v.b.\rangle$ Bloch functions at the R-point of the Brillouin zone are given by:

$$p_x = \langle c.b., x|\hat{p}_x|v.b.\rangle, \quad p_y = \langle c.b., y|\hat{p}_y|v.b.\rangle, \quad p_z = \langle c.b., z|\hat{p}_z|v.b.\rangle. \tag{8}$$

A representation of the wave functions is given in Ref. [1].

Pump excitation with circular polarization leads to creation of an exciton. However, as the excitons have short lifetimes ($\approx 160\,\text{ps}$), the pump-probe signal at a nanosecond time scale is provided by resident carrier spins localized in the NCs. We assume that the NCs, being initially uncharged, become charged through the optical excitation, where one of the charges becomes trapped in the NC. This photocharging is long living, and when the next optical pulse generates an additional exciton, this leads to formation of a trion complex with the resident carrier. Supplementary Figures 9b,c show level schemes of the photogeneration of negative and positive trions in a nanocrystal by σ^+ polarized excitation. If uncharged, σ^+ polarized light excites in the NC the conduction band state with wave function $c[+1/2]$

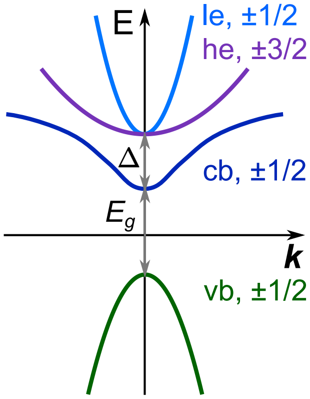

Supplementary Figure 8. **Schematics of perovskite band structure.** The spin ±1/2 valence band (vb) and the spin ±1/2 conduction band (cb) are separated by the optical band gap (E_g). Above the cb the heavy-electron (he) and light-electron (le) bands with spin ±3/2 and ±1/2, respectively, are located, set apart by the spin-orbit energy (Δ).

and the valence band state with wave function $v[+1/2]$, marked by $|\uparrow\rangle$ and $|\Uparrow\rangle$, respectively. If the nanocrystal contains a resident electron with wave function $c[-1/2]$, marked by $|\downarrow\rangle$, the light creates a negatively charged trion (T^-). In the case of a resident hole with a wave function $v[-1/2]$, marked by $|\Downarrow\rangle$, a positively charged trion (T^+) is generated.

An optical pulse leads to the formation of trions, which capture electrons (or holes) with a specific spin orientation. These carriers do not contribute to the spin polarization, as the trion states form singlets. The fraction of electrons that is not captured into the trions becomes polarized. Thus, an initially unpolarized ensemble of resident carriers becomes polarized, at least at timescales shorter than the trion lifetime. Under certain conditions, the carriers returning after trion recombination do not restore the initial unpolarized state of an ensemble. This takes place for a fast trion spin relaxation or fast electron Larmor precession of the remaining (uncaptured) spins [18, 19].

The matrix elements of the optical transitions created by the circularly polarized light from the electron states to the negative trion states are taken from the Ref. [20], and for the negative trion (T^-) can be written as:

$$M_{T^-}(c[-1/2]; v[+1/2]) \propto -\frac{\cos\xi}{\sqrt{2}}(p_{x'}E_{x'} - ip_{y'}E_{y'}) \propto d_1E_1, \tag{9}$$

$$M_{T^-}(c[+1/2]; v[-1/2]) \propto -\frac{\cos\xi}{\sqrt{2}}(p_{x'}E_{x'} + ip_{y'}E_{y'}) \propto d_2E_2, \tag{10}$$

$$M_{T^-}(c[-1/2]; v[-1/2]) \propto -\sin\xi E_{z'}p_{z'} \propto d_3E_3, \tag{11}$$

$$M_{T^-}(c[+1/2]; v[+1/2]) \propto \sin\xi E_{z'}p_{z'} \propto -d_3E_3. \tag{12}$$

One should note that, for the σ^+ circularly polarized light $E_x = iE_y$ in the laboratory coordinate system, then $E_{x'} \pm iE_{y'} = E_x \exp(\mp i\phi)(\cos\theta \mp 1)$ in the NC coordinate system.

If $p_{x'} = p_{y'} = p_\perp \neq p_z = p_\parallel$, one can rewrite matrix elements for σ^+ polarized light:

$$d_1E_1 \propto -\frac{\cos\xi p_\perp}{\sqrt{2}}E_x \exp(i\phi)(\cos\theta + 1), \tag{13}$$

$$d_2E_2 \propto -\frac{\cos\xi p_\perp}{\sqrt{2}}E_x \exp(-i\phi)(\cos\theta - 1), \tag{14}$$

$$d_3E_3 \propto -\sin\xi p_\parallel E_x \sin\theta. \tag{15}$$

In the cubic approximation $\sin\xi = 1/\sqrt{3}$, $\cos\xi = \sqrt{2/3}$ and $p_\perp = p_\parallel = p$.

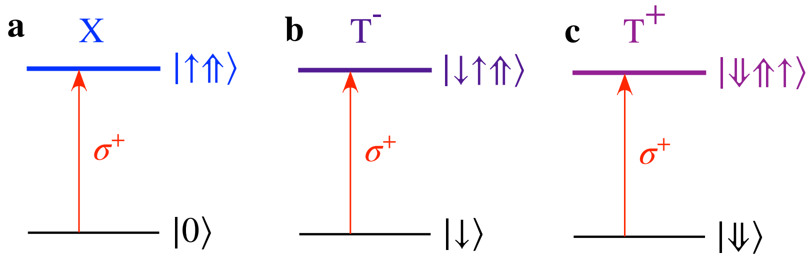

Supplementary Figure 9. Optical transitions for the generation by a σ^+ polarized photon of: (**a**) an exciton , (**b**) T^- , and (**c**) T^+. $|\uparrow\rangle$ and $|\downarrow\rangle$ indicate the electron states with the wave functions $c[+1/2]$ and $c[-1/2]$, respectively. $|\Uparrow\rangle$ and $|\Downarrow\rangle$ indicate the hole states with the wave functions $v[+1/2]$ and $v[-1/2]$, respectively. The trions are photogenerated in their ground state, which is a spin singlet of the electrons or holes.

The matrix elements of the optical transitions to a positive trion (T^+) are:

$$M_{T^+}(v[-1/2];c[+1/2]) = M_{T^-}(c[-1/2];v[+1/2]) \propto d_1E_1, \tag{16}$$

$$M_{T^+}(v[+1/2];c[-1/2]) = M_{T^-}(c[+1/2];v[-1/2]) \propto d_2E_2, \tag{17}$$

$$M_{T^+}(v[+1/2];c[+1/2]) = M_{T^-}(c[-1/2];v[-1/2]) \propto d_3E_3, \tag{18}$$

$$M_{T^+}(v[-1/2];c[-1/2]) = M_{T^-}(c[+1/2];v[+1/2]) \propto -d_3E_3. \tag{19}$$

The Hamiltonian of the interaction of the nanocrystal with light in the basis ($c[1/2]$; $c[-1/2]$; $v[1/2]$; $v[-1/2]$) for T^- creation is represented by:

$$\hat{H} = \frac{\hbar}{2}\begin{pmatrix} 0 & 0 & -f_3^* e^{i\omega t} & f_2^* e^{i\omega t} \\ 0 & 0 & f_1^* e^{i\omega t} & f_3^* e^{i\omega t} \\ -f_3 e^{-i\omega t} & f_1 e^{-i\omega t} & 0 & 0 \\ f_2 e^{-i\omega t} & f_3 e^{-i\omega t} & 0 & 0 \end{pmatrix}. \tag{20}$$

Here $\hbar$ is the reduced Planck constant, ω is the optical frequency of the light, t is the time, $f_i(t) = -\frac{2e^{i\omega t}}{\hbar}\int d_i(\mathbf{r})E_i(\mathbf{r},t)d^3r$ is the time-dependent matrix element for description of the light interaction with a nanocrystal with index $i = (1,2,3)$. For T^+ in the basis ($v[1/2]$; $v[-1/2]$; $c[1/2]$; $c[-1/2]$) one needs to change the sign of f_3 in the Hamiltonian (20). One can introduce the analytical expressions for the matrix element describing the interaction with a σ^+ circularly polarized optical pulse with duration τ_p and pulse area Θ for the simplified case $p_{x'} = p_{y'} = p_\perp \neq p_z = p_\parallel$:

$$f_1\tau_p \propto -\frac{\Theta}{2}e^{i\phi}(\cos\theta + 1), \tag{21}$$

$$f_2\tau_p \propto \frac{\Theta}{2}e^{-i\phi}(\cos\theta - 1), \tag{22}$$

$$f_3\tau_p \propto \frac{\Theta}{2}\alpha\sin\theta. \tag{23}$$

Here $\alpha = p_\parallel\sqrt{2}\tan\xi/p_\perp$, in cubic approximation $\alpha = 1$. Therefore, a short pulse with rectangular shape changes the wave functions ψ_1^b and ψ_2^b of the electron in the conduction band $c[1/2]$ and $c[-1/2]$, respectively, to the wave functions after pulse action ψ_1^a and ψ_2^a. Here index 'b' stands for 'before' and 'a' for 'after' pulse action:

$$\psi_1^a = \frac{e^{i\omega'\tau_p/2}}{2}\left[\psi_1^b\left(K_p\left[1+\frac{\Delta}{W}\right] + K_m\left[1-\frac{\Delta}{W}\right]\right) + \psi_2^b\frac{2\Pi}{W}(K_p - K_m)\right], \tag{24}$$

$$\psi_2^a = \frac{e^{i\omega'\tau_p/2}}{2}\left[\psi_2^b\left(K_m\left[1+\frac{\Delta}{W}\right]+K_p\left[1-\frac{\Delta}{W}\right]\right)+\psi_1^b\frac{2\Pi^*}{W}(K_p-K_m)\right]. \quad (25)$$

$\omega' = \omega_p - \omega_0$ is the optical detuning between the pulse central frequency ω_p and the trion resonant frequency ω_0. Δ and Π are coefficients composed from f_i:

$$\Delta = \frac{1}{4}(|f_2|^2 - |f_1|^2) = -\frac{|\Theta|^2}{4}\cos\theta, \quad (26)$$

$$\Pi = \frac{1}{4}(-f_1^* f_3 + f_2 f_3^*) = \mp\frac{|\Theta|^2}{8}\alpha\sin\theta\exp(-i\phi). \quad (27)$$

Here the expressions on the right are written for the simplified case $p_{x'} = p_{y'} = p_\perp \neq p_z = p_\parallel$. The upper sign in Eq. (27) corresponds to T^-, and the lower one corresponds to T^+.

$$W = \sqrt{\Delta^2 + 4|\Pi|^2}, \quad (28)$$

$$K_{p,m} = \cos\left(\frac{\Omega_{p,m}\tau_p}{2}\right) - \frac{i\omega'}{\Omega_{p,m}}\sin\left(\frac{\Omega_{p,m}\tau_p}{2}\right), \quad (29)$$

$$\Omega_{p,m} = \sqrt{(\omega')^2 + \frac{1}{2}(|f_1|^2 + |f_2|^2 + 2|f_3|^2) \pm 2W}. \quad (30)$$

Ω corresponds to the Rabi oscillation frequency. In the cubic approximation $\Omega\tau_p = \sqrt{(\omega'\tau_p)^2 + \Theta^2}$.

A short circularly polarized pump pulse changes the resident hole spin polarization components in a single NC according to the equations:

$$\mathbf{S}^a = \mathcal{A}\cdot\mathbf{S}^b + \mathbf{S}_0 \quad (31)$$

$$\mathcal{A} = \begin{pmatrix} \mathcal{A}_{11} & \mathcal{A}_{12} & \mathcal{A}_{13} \\ \mathcal{A}_{21} & \mathcal{A}_{22} & \mathcal{A}_{23} \\ \mathcal{A}_{31} & \mathcal{A}_{32} & \mathcal{A}_{33} \end{pmatrix}, \quad (32)$$

$$\mathbf{S}_0 = \begin{pmatrix} S_{x',0} \\ S_{y',0} \\ S_{z',0} \end{pmatrix}, \qquad \mathbf{S}^a = \begin{pmatrix} S^a_{x'} \\ S^a_{y'} \\ S^a_{z'} \end{pmatrix}, \qquad \mathbf{S}^b = \begin{pmatrix} S^b_{x'} \\ S^b_{y'} \\ S^b_{z'} \end{pmatrix}. \quad (33)$$

$\mathbf{S}^a$ is the spin polarization after the pulse arrival, $\mathbf{S}^b$ is the spin polarization before the pulse arrival.

$$\mathcal{A}_{11} = [|K_p + K_m|^2 - \frac{(\Delta^2 - 4\mathrm{Re}(\Pi^2))}{W^2}|K_p - K_m|^2]/4, \quad (34)$$

$$\mathcal{A}_{12} = \frac{\Delta}{W}\mathrm{Im}(K_p K_m^*) - \frac{\mathrm{Im}(\Pi^2)}{W^2}|K_p - K_m|^2, \quad (35)$$

$$\mathcal{A}_{13} = \frac{2\mathrm{Im}(\Pi)}{W}\mathrm{Im}(K_p K_m^*) + \frac{\Delta}{W^2}\mathrm{Re}(\Pi)|K_p - K_m|^2, \quad (36)$$

$$\mathcal{A}_{21} = -\frac{\Delta}{W}\mathrm{Im}(K_p K_m^*) - \frac{\mathrm{Im}(\Pi^2)}{W^2}|K_p - K_m|^2, \quad (37)$$

$$\mathcal{A}_{22} = [|K_p + K_m|^2 - \frac{(\Delta^2 + 4\mathrm{Re}(\Pi^2))}{W^2}|K_p - K_m|^2]/4, \quad (38)$$

$$\mathcal{A}_{23} = \frac{2\Re(\Pi)}{W}\mathrm{Im}(K_p K_m^*) - \frac{\Delta}{W^2}\mathrm{Im}(\Pi)|K_p - K_m|^2, \tag{39}$$

$$\mathcal{A}_{31} = \frac{-2\mathrm{Im}(\Pi)}{W}\mathrm{Im}(K_p K_m^*) + \frac{\Delta}{W^2}\mathrm{Re}(\Pi)|K_p - K_m|^2, \tag{40}$$

$$\mathcal{A}_{32} = -\frac{2\mathrm{Re}(\Pi)}{W}Im(K_p K_m^*) - \frac{\Delta}{W^2}\mathrm{Im}(\Pi)|K_p - K_m|^2, \tag{41}$$

$$\mathcal{A}_{33} = [|K_p + K_m|^2 + \frac{(\Delta^2 - 4|\Pi|^2)}{W^2}|K_p - K_m|^2]/4, \tag{42}$$

$$S_{x',0} = \frac{\mathrm{Re}(\Pi)}{2W}(|K_p|^2 - |K_m|^2), \tag{43}$$

$$S_{y',0} = -\frac{\mathrm{Im}(\Pi)}{2W}(|K_p|^2 - |K_m|^2), \tag{44}$$

$$S_{z',0} = \frac{\Delta}{4W}(|K_p|^2 - |K_m|^2). \tag{45}$$

Eqs. (31-45) describe the generation of the electron and hole spin polarization by a short optical pulse in a single NC with random c-axis orientation.

SUPPLEMENTARY NOTE 7: SPIN DYNAMICS IN EXTERNAL MAGNETIC FIELD

Here we describe the dynamics of the spin polarization in an external magnetic field. In a single NC, the hole or electron spin oriented by a circularly polarized pump pulse precesses about the external magnetic field, which can be described using a semiclassical approach [21]:

$$\frac{d\mathbf{S}}{dt} = [\boldsymbol{\omega} \times \mathbf{S}]\,. \tag{46}$$

Here $\boldsymbol{\omega} = (\omega_{x'}, \omega_{y'}, \omega_{z'})$ is the Larmor precession frequency. $\omega_i = \mu_B g_i B_i/\hbar$, here $i = (x', y', z')$ and $\boldsymbol{g} = (g_{x'}, g_{y'}, g_{z'})$ is the anisotropic g-factor. The magnetic field components on the axis of the nanocrystal for an orientation $\mathbf{B} \parallel x$ in the laboratory coordinate system are given by:

$$B_{x'} = B_x \cos\theta \cos\phi, \tag{47}$$
$$B_{y'} = -B_x \cos\theta \sin\phi, \tag{48}$$
$$B_{z'} = B_x \sin\theta. \tag{49}$$

Eq. (46) is written in a form that includes spin relaxation for an anisotropic g-factor in a single NC:

$$\mathbf{S}(t) = \mathcal{B}(t) \cdot \mathbf{S}^a, \tag{50}$$

$$\mathcal{B}(t) = \begin{pmatrix} \mathcal{B}_{11} & \mathcal{B}_{12} & \mathcal{B}_{13} \\ \mathcal{B}_{21} & \mathcal{B}_{22} & \mathcal{B}_{23} \\ \mathcal{B}_{31} & \mathcal{B}_{32} & \mathcal{B}_{33} \end{pmatrix} \cdot \exp(-t/\tau_s), \tag{51}$$

$$\mathcal{B}_{11} = \cos(\omega_{\mathrm{L}} t) + \frac{g_x^2}{\tilde{g}^2}\cos^2\theta\cos^2\phi[1 - \cos(\omega_{\mathrm{L}} t)], \tag{52}$$

$$\mathcal{B}_{12} = -\left[\frac{g_x g_y}{2\tilde{g}^2}\cos^2\theta\sin 2\phi\left[1-\cos(\omega_L t)\right] + \frac{g_z}{\tilde{g}}\sin\theta\sin(\omega_L t)\right], \tag{53}$$

$$\mathcal{B}_{13} = \frac{g_x g_z}{2\tilde{g}^2}\cos\phi\sin 2\theta\left[1-\cos(\omega_L t)\right] - \frac{g_y}{\tilde{g}}\sin\phi\cos\theta\sin(\omega_L t), \tag{54}$$

$$\mathcal{B}_{21} = \frac{g_z}{\tilde{g}}\sin\theta\sin(\omega_L t) - \frac{g_x g_y}{2\tilde{g}^2}\cos^2\theta\sin 2\phi\left[1-\cos(\omega_L t)\right], \tag{55}$$

$$\mathcal{B}_{22} = \cos(\omega_L t) + \frac{g_y^2}{\tilde{g}^2}\cos^2\theta\sin^2\phi\left[1-\cos(\omega_L t)\right], \tag{56}$$

$$\mathcal{B}_{23} = -\frac{g_z g_y}{2\tilde{g}^2}\sin\phi\sin 2\theta\left[1-\cos(\omega_L t)\right] - \frac{g_x}{\tilde{g}}\cos\theta\cos\phi\sin(\omega_L t), \tag{57}$$

$$\mathcal{B}_{31} = \frac{g_z g_x}{2\tilde{g}^2}\cos\phi\sin 2\theta\left[1-\cos(\omega_L t)\right] + \frac{g_y}{\tilde{g}}\cos\theta\sin\phi\sin(\omega_L t), \tag{58}$$

$$\mathcal{B}_{32} = \frac{g_z g_y}{2\tilde{g}^2}\sin\phi\sin 2\theta\left[1-\cos(\omega_L t)\right] + \frac{g_x}{\tilde{g}}\cos\theta\cos\phi\sin(\omega_L t). \tag{59}$$

$$\mathcal{B}_{33} = 1 - \frac{g_\perp^2}{\tilde{g}^2}\cos^2\theta\left[1-\cos(\omega_L t)\right]. \tag{60}$$

$\mathbf{S}(t)$ contains the time-dependent components of the electron or hole spin polarization. $\omega_L = \mu_B\tilde{g}B/\hbar$ is the Larmor precession frequency in the external magnetic field. $\tilde{g} = \sqrt{g_\perp^2\cos^2\theta + g_z^2\sin^2\theta}$ is the effective g-factor. $g_\perp = \sqrt{g_x^2\cos^2\phi + g_y^2\sin^2\phi}$. τ_s is the spin relaxation time. Here, the isotropic spin relaxation time is $\tau_s = T_2$. Also, we assume that the trion spin relaxation is faster than the spin relaxation of the resident carrier and since g-factors of the resident carrier and the unpaired carrier in the trion differ significantly, we can neglect the polarization "returning from the trion". As a result, the resident carrier gets polarized right after the trion is formed.

In the experiment, the pump pulse creates a spin polarization, which subsequently precess in the external magnetic field. If the carrier spins do not loose the polarization until the arrival of the next pulse, i.e. for $T_2 > T_R$, then spin polarization is accumulated. It is possible to model the experiment by considering an infinite number of pulses with subsequent dynamics in the magnetic field [22]:

$$\mathbf{S}^a = (\mathbf{I} - \mathcal{A}\mathcal{B})^{-1}\mathbf{S}_0, \tag{61}$$

with $\mathbf{I}$ being the unity matrix.

SUPPLEMENTARY NOTE 8: DISTRIBUTION OF ELECTRON (HOLE) LARMOR PRECESSION FREQUENCY IN ENSEMBLE OF NANOCRYSTALS

The dispersion of g-factors (Δg) and nuclear spin fluctuations ($\Delta\omega_N = g\mu_B\Delta B/\hbar$) broadens the spectrum of Larmor frequencies in an ensemble of nanocrystals [22]. The resulting spectral distribution can be described by a Gaussian function:

$$\rho(\omega_L) = \exp\left[-\frac{(\omega_L - \omega_{L,0})^2}{2(\Delta\omega)^2}\right]. \tag{62}$$

Here, $\omega_{L,0}$ is the central Larmor frequency of the distribution and $\Delta\omega = \sqrt{(\Delta g\mu_B B_V/\hbar)^2 + (\Delta\omega_N)^2}$ is the frequency dispersion. In strong external magnetic fields exceeding the exchange field of the nuclear spin fluctuations ($B_V \gg \Delta B$) $\Delta\omega \approx \Delta g\mu_B B_V/\hbar$. Obviously, for electrons and holes $\Delta\omega$ and ΔB are different, as they have different g-factors and Δg, as well as different hyperfine constants describing their interaction with the nuclei [1].

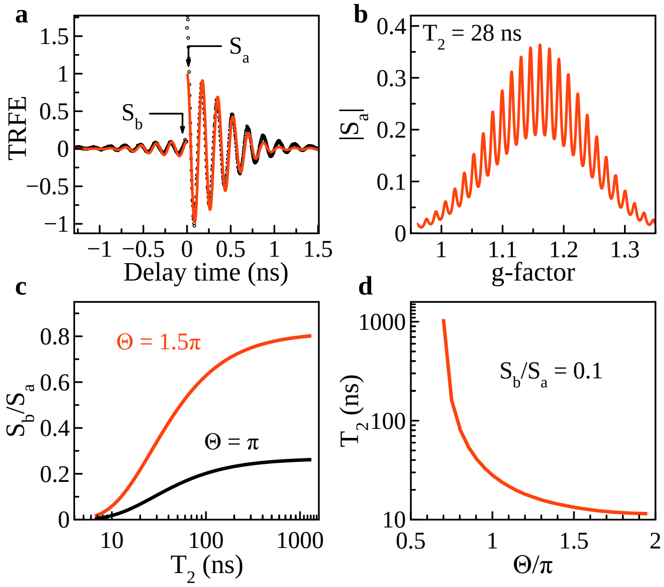

Supplementary Figure 10. **Modeling of spin dynamics without account for NIFF. a**, Time-resolved Faraday ellipticity signal (black dots) measured at $B_{\rm V} = 0.35\,$T and its modeling (red line) using parameters: $T_{\rm R} = 13.2\,$ns, $\Theta = \pi$, $\Delta\omega = 2.5\,$rad/ns, $T_2 = 28\,$ns, and $\omega' = 0$. **b**, Spectral distribution of precessing modes for $T_2 = 28\,$ns. **c**, S_b/S_a dependence on T_2 for $\Theta = \pi$ (black line) and $\Theta = 1.5\pi$ (red line). **d**, Relation between the spin coherence time T_2 and the pulse power Θ for a fixed value of the amplitude ratio $S_b/S_a = 0.1$, as observed in the experiment.

SUPPLEMENTARY NOTE 9: MODELING OF SML IN PEROVSKITE NCS

The simulation of the spin dynamics is carried out in four steps:
1) For a single NC the spin polarization components are calculated after the action of a pump pulse.
2) The precession of the spin polarization in an external magnetic field is calculated for a single NC.
3) The polarization components after action of an infinitely long pulse sequence are calculated.
4) Steps $1-3$ are repeated for all possible precession frequencies in the nanocrystal ensemble with certain g-factor dispersion.

Without loss of generality we consider resident holes in the NCs, that are created by photocharging. The pump-probe Faraday ellipticity signal is generated through a trion intermediate state for the resident hole spin polarization (details of spin coherence generation are given in Supplementary Note 6). The spin polarization components tilted relative to the direction of the external magnetic field precess in time and decay with the spin coherence time T_2. If $T_2 \geq T_R$, then the spin polarization accumulates being excited by an infinite sequence of optical pulses, as in the experiment (for details see Supplementary Note 7).

The SML is an ensemble effect being formed by adding a large number of oscillating signals with frequencies commensurate with the repetition rate of the laser pulses and $T_2 \geq T_{\rm R}$. Therefore, the model takes into account the spread of the g-factors (Supplementary Note 8). Numerical modeling has been performed according to Supplementary Eqs. (26-62). The simulated spin polarization (S_a) distribution after an infinite number of optical pulses at $B_{\rm V} = 0.35\,$T is shown in Fig. 10b. The width of the distribution is defined by $2\Delta g$ with $\Delta g = 0.1$, the multiple peaks correspond to precession modes, and the width of each peak is determined by $1/T_2$. For simplicity we assume that the spin coherence time is isotropic and the same for all precession modes. The SML signal corresponding to the distribution is shown by red line in Fig. 10a compared to the experimental dynamics (black dots). The signal decay for negative and positive time delays is defined by Δg. The ratio S_b/S_a of the amplitudes before pulse arrival S_b and after pulse arrival S_a is determined by T_2, the optical pulse area Θ and also the NIFF effect which will be considered below in Supplementary Note 10. The numerical calculations show that integration over the angles ϕ and θ changes $\mathbf{S}_a$ and $\mathbf{S}_b$ in the same way. Therefore, the SML amplitude in case of cubic symmetry and the relative value S_b/S_a are independent of the random orientation of the crystals. The value S_b/S_a is, however, sensitive to the Rabi frequency $\Omega\tau_p = \sqrt{(\omega'\tau_p)^2 + \Theta^2}$. It also depends on the optical detuning ω' and the pump pulse duration τ_p, but does not depend on ϕ and θ. The Larmor frequency depends on the magnitude of the external magnetic field, but not on its

direction.

As can be seen in Fig. 10c, S_b/S_a strongly depends on the spin coherence time T_2 due to efficiency of the spin amplification effect and on the pulse area Θ due to the Rabi frequency. The S_b/S_a value increases for spin systems with longer T_2. The dependence of the S_b/S_a value on T_2 reaches saturation with a value depending on the pump pulse area. Note that $\Theta = \pi$ is not optimal for the SML effect, despite that it is the optimal condition for spin coherence generation as explained in Ref. 9.

Supplementary Figure 10d shows the range of T_2 and Θ for which the value of $S_b/S_a = 0.1$ is taken as in the experiment. It can be obtained for $\Theta \in [0.7\pi; 2\pi]$ with T_2 varying from 10 ns to 1 μs. There is an experimental way to estimate the upper limit of T_2 time, namely to increase the period between laser pulses and reach the regime when spin coherence is fully decayed before the next pump pulse arrival. In this case the SML signal amplitude at negative time delay should vanish. In Fig. 11 we show TRFE dynamics measured with a use of pulse-picker, which allows us to increase the laser repetition period up to $T_{\mathrm{R}} = 39.6$ ns. The SML signal is not detectable setting an upper limit for $T_2 < 40$ ns. For such rather short time the SML signal generation is possible only at high pumping intensities. For $\Theta = \pi$ the hole spin coherence time of $T_2 = 28$ ns can be evaluated.

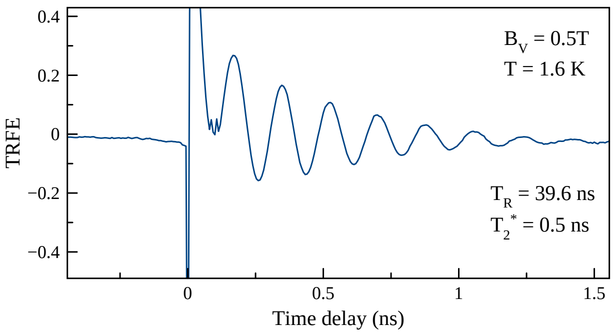

Supplementary Figure 11. **Time-resolved Faraday ellipticity measured at lower repetition rate.** Time-resolved Faraday ellipticity signal measured at $B_{\mathrm{V}} = 0.5$ T and $T = 1.6$ K for $T_{\mathrm{R}} = 39.6$ ns, pump power is 3.3 mW.

SUPPLEMENTARY NOTE 10: EVALUATION OF SPIN COHERENCE TIME IN THE PRESENCE OF NIFF

Determination of the spin coherence and spin dephasing times also requires an estimate of their value in the presence of nuclei-induced frequency focusing (NIFF). After the experimental observation of the NIFF effect in (In,Ga)As quantum dots, a number of models were proposed to describe it [23, 26–30]. Among them, the model of dynamic nuclear polarization is the most physically transparent and universal one; it describes most of the observed experimental effects in various semiconductor structures [31–33]. This model considers nonresonant optical pumping of the hole or electron spin ensemble and dynamic nuclear polarization arising due to the transfer of the charge carrier spin to the ensemble of nuclei via the hyperfine interaction. Such flip-flop processes are most efficient in the presence of an electron or hole spin component along the external magnetic field. It was noted in Ref. [27] (see also [22]) that the corresponding spin component S_x along the magnetic field $\mathbf{B}_{\mathrm{V}} \parallel x$ can appear in our geometry taking into account the charge carrier spin rotation by the light pulse: In this case, there is an optical Stark field resulting in an effective magnetic field acting on the resident carrier spins that is directed along the wave vector of light. Due to the precession in this effective field, the spins gain a polarization projection (S_x) along the direction of the external magnetic field. The dynamics of nuclear polarization I_{N} along the magnetic field is described by the kinetic equation:

$$\frac{dI_{\mathrm{N}}}{dt} + \frac{1}{T_{1\mathrm{h}}}[I_{\mathrm{N}} - \bar{Q}\langle S_x(I_{\mathrm{N}})\rangle] + \frac{I_{\mathrm{N}}}{T_{\mathrm{d}}} = 0. \tag{63}$$

Here $\bar{Q} = 4I(I+1)/3$ is a factor that depends on the nuclear spin I and $\bar{Q} = 1$ for perovskite NCs with $I(^{207}\mathrm{Pb}) = 1/2$, $\langle S_x(I_{\mathrm{N}})\rangle$ is the average spin polarization of the resident charge carrier along the magnetic field axis found from the solution of Eqs. (26-61), $T_{1\mathrm{h}}$ is the hyperfine coupling induced spin-flip time and T_{d} is the nuclear spin-lattice relaxation

time, which takes into account any other possible spin-leakage mechanisms. $T_{\rm d} = 0.5\,$s is determined from experimental data shown in Fig. 2a in the main text. In the quasi-steady state conditions where the pulse train duration exceeds by far the nuclear relaxation times $dI_{\rm N}/dt$ can be neglected. The relative magnitude of these times can conveniently be summarized in the leakage factor $f_{\rm N} = T_{\rm d}/(T_{\rm 1h} + T_{\rm d})$. The nuclear polarization produces a nonzero Overhauser field $B_{\rm N} = \alpha A_h I_{\rm N}/\mu_{\rm B} g_h$, which acts back on the carrier spins. For our case, the hyperfine constant for the hole spins with the ^{207}Pb isotope having a natural abundance of $\alpha = 0.22$ is $A_h = 33\,\mu$eV Ref. [1]. The nuclear polarization contributes to the total magnetic field $B_{\rm V} + B_{\rm N}$, providing a feedback on the hole spin precession with the effective frequency $\omega_{\rm L} = \mu_{\rm B} g_h (B_{\rm V} + B_{\rm N})/\hbar$. Correspondingly, the hole-nuclear spin-flip rate can be estimated as

$$\frac{1}{T_{\rm 1h}} \propto \left(\frac{A_h}{\hbar N}\right)^2 \frac{2F\tau_{\rm c}}{1 + \omega_{\rm L}^2 \tau_{\rm c}^2}, \tag{64}$$

and depends on the nuclear polarization via $\omega_{\rm L}$. Here N is the number of unit cells within the hole orbit, for perovskite NCs $N = 10^4$ [1], $\tau_{\rm c}$ is the correlation time in the hole-nuclear spin system, the factor F is the probability of finding the hole at the localization site. $\tau_{\rm c} = 13\,$ns is the parameter chosen to fulfill the ratio of $\omega_{\rm L}\tau_{\rm c} \gg 1$ and $F = 1$ is taken as the fitting parameter. Details of the calculation and extended equations can be found in Ref. [33].

The calculated Overhauser field distribution for perovskite NCs is shown in Fig. 12a in dependence on ω_x/ω_R. The Overhauser field is $B_{\rm N} = 0$ when ω_x is commensurate with $\omega_{\rm R}$ and is maximal for hole spins precessing with frequencies commensurate with $\omega_R/2$. Therefore, all precession frequencies lying between integer values $\omega_x/\omega_{\rm R}$ are dragged towards the precession modes. The nonlinearity in the system, resulting from the dependence of the hole spin precession frequency on the nuclear polarization itself, results in a variation of the nuclear spin-flip rates which, in turn, affects the build-up of the nuclear spin polarization.

As a result, a non-equilibrium distribution of nuclear fields is produced. An increase of the nuclear polarization results in suppression of nuclear spin fluctuations magnitude [31, 34] and extension of the resident carrier spin dephasing time:

$$\mathcal{T}_2^* \propto \frac{\hbar}{\mu_B g_h \sqrt{\langle \delta B_{\rm N}^2 \rangle}}. \tag{65}$$

One should note that suppression of the nuclear spin fluctuations lead to distribution of the spin coherence time around the mode position, therefore we define it by the homogeneous dephasing time $\mathcal{T}_2^*$. In the main text and

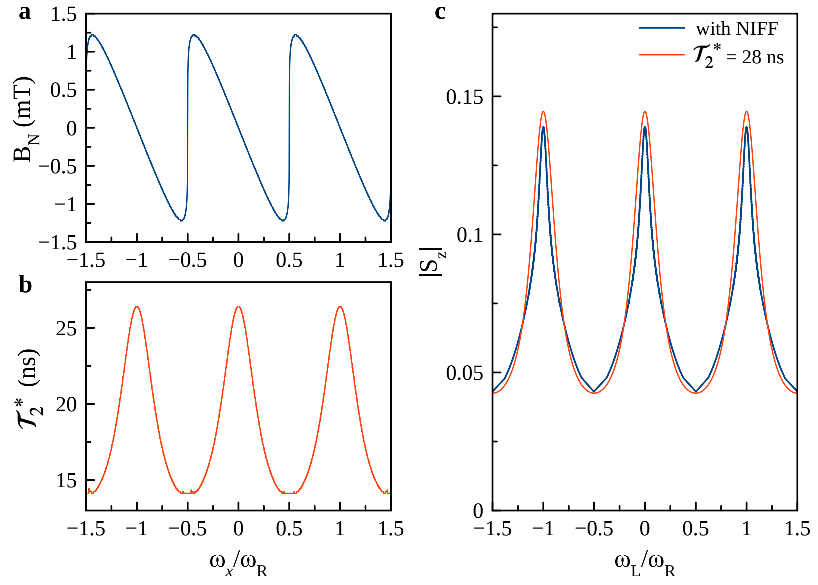

Supplementary Figure 12. **Nuclei-induced frequency focusing effect.** **a**, Calculated Overhauser field distribution as a function of $\omega_x/\omega_{\rm R}$ using the parameters $T_{\rm R} = 13.2\,$ns, $\Theta = \pi$, $\mathcal{T}_{2,0}^* = 13\,$ns, $I(^{207}{\rm Pb}) = 1/2$, $A_h = 33\,\mu$eV, $\alpha = 0.22$, 10^4 nuclear spins, correlation time of the hole-nuclei spin interactions $\tau_c = 13\,$ns, $T_d = 0.5\,$s and $\omega' \tau_p/2\pi = 0.41$. **b**, $\mathcal{T}_2^*$ modulation in the presence of NIFF. **c**, Hole spin polarization distribution calculated for $\mathcal{T}_2^* = 28\,$ns without NIFF (red line), and in the presence of NIFF for $\mathcal{T}_{2,0}^* = 13\,$ns (blue line).

in the Supplementary Note 9, the distribution of the spin coherence time around the mode position is not taken into account, so that the mode width is defined by T_2 and the width of the precession frequency distribution in the ensemble determines the inhomogeneous dephasing time T_2^*.

As suggested in Ref. [31], for the carrier spins satisfying the PSC the strong feedback should lead to a reduction of the nuclear spin fluctuations. As soon as ω_x/ω_R differs from K, the nuclear fluctuations recover due to the reduced feedback strength. Therefore, depending on the magnetic field, the spin dephasing time becomes strongly modulated due to the periodic changes of the amplitude of the nuclear fluctuations.

We calculated the spin dephasing time $\mathcal{T}_2^*$ dependence on the magnetic field with the parameters relevant for the studied $CsPb(Cl,Br)_3$ NCs and plotted it in Fig. 12b as a function of ω_x/ω_R. The modulation of $\mathcal{T}_2^*$ around the precession mode is clearly seen. Here, we have used the fitting parameter $\mathcal{T}_{2,0}^* = 13$ ns for the maximal nuclear spin fluctuations magnitude at $\omega_x/\omega_R = 1/2 + K$, where K is an integer. The suppression of nuclear spin fluctuations leads to a prolongation of the spin dephasing time of the hole spins precessing on $\omega_x/\omega_R = K$ as shown in Fig. 12b.

Supplementary Figure 12c shows a calculated distribution near $\omega_x/\omega_R = 0$ and ± 1 to account for the spin dephasing time modulation in the dynamic nuclear polarization mechanism. The initial spin dephasing time is defined by the maximal magnitude of the nuclear spin fluctuations $\mathcal{T}_{2,0}^* = 13$ ns, which becomes then extended by the reduced nuclear fluctuation at the modes. Since the calculation is numerical, we cannot give an analytical equation for the peak width, but it is comparable to the peak width for the hole polarization calculated without the NIFF effect, or for constant $\mathcal{T}_2^* = T_2 = 28$ ns.

To summarize, the effect of suppression of nuclear spin fluctuations in the dynamic nuclear polarization mechanism leads to an effective prolongation of the spin coherence time on the precession modes. This means that for a constant ratio of the mode-locking amplitudes $S_b/S_a = 0.1$ the spin coherence time extracted from the model is shorter if the NIFF effect is taken into account, $\mathcal{T}_{2,0}^* = T_2 = 13$ ns, and longer if it is neglected, $T_2 = 28$ ns.

SUPPLEMENTARY NOTE 11: SAMPLE INFORMATION

XRD evaluation of NCs sizes

The studied $CsPb(Cl,Br)_3$ nanocrystals embedded in fluorophosphate $Ba(PO_3)_2$ glass were synthesized by rapid cooling of a glass melt enriched with the components needed for the perovskite crystallization. Samples of fluorophosphate (FP) glasses of $60Ba(PO_3)_2-15NaPO_3-12AlF_3-1Ga_2O_3-4Cs_2O{-}8PbF_2$ (mol. %) composition doped with 16 mol.% NaCl, 3.4 mol.% $BaBr_2$ were prepared using the melt-quench technique. The glass synthesis was performed

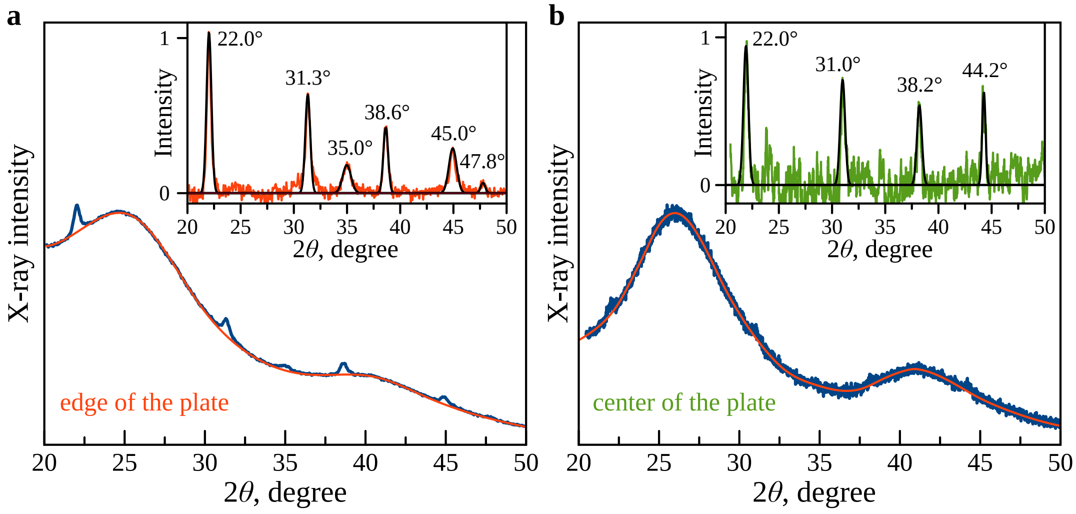

Supplementary Figure 13. **X-ray diffraction patterns from NCs.** XRD patterns for two positions on the glass plate: **a**, the edge with the larger NCs, and **b**, the center with the smaller NCs. Measurements are performed at a room temperature. The red lines are the spline fits. The inserts demonstrate the signal normalized to the amplitude of the first peak after the subtraction of spline. The numbers above the peaks indicate the double Bragg angle, 2θ. The black lines are the fit with the Gaussian.

in a closed glassy carbon crucible at temperatures of 1000-1050°C. About 50 g of the mixed powder was melted in the crucible for 20 min. Then the glass melt was cast on a glassy carbon plate and pressed to form a plate with a thickness of about 2 mm. The carbon plate has an inhomogeneous cooling rate, so its center is colder than the edge. Therefore, the growth of NCs stops in the center of the plate earlier than at the edges, which leads to a formation of a smaller NCs in the center. The sample studied in this paper was taken from the middle of a glass plate.

The X-ray diffraction (XRD) pattern of the sample with large NCs measured at room temperature in the Rigaku X-ray diffractometer is shown in Fig. 13a. A set of X-ray diffraction scattering peaks are observed in the pattern. To extract the peak positions and their width we fit the XRD curve with a spline function and subtract it to remove the background variation. The resulting peaks, shown by the insert, can be approximated by a Gaussian functions with peak positions at the Bragg angles (2θ) indicated above each peak and the full-width at half-maxima β. Peak parameters determine the average NCs diameter d as:

$$d = \frac{K\lambda}{\beta \cos\theta}. \tag{66}$$

Here $K = 0.9$ is a dimensionless shape factor. $\lambda = 0.154$ nm is the X-ray wavelength. The diameter is calculated for each peak and then averaged, leading to $d = (14 \pm 1)$ nm. This calculation should give the upper limit for the smaller NCs used in our work. The XRD pattern for the sample part from the center of the glass plate is shown in Fig. 13b. The insert signal demonstrates similarly narrow peak structures as in the insert to the panel Fig. 13a, which can be assigned to a residual fraction of large NC. The XRD method turns out to be less efficient for the smaller NCs and the decreased signal-to-noise ratio does not allow us to resolve wider peak structures. Therefore, to confirm the presence of NCs with sizes smaller than 14 nm we shall turn our attention to an alternative method, to transmission electron microscopy (TEM).

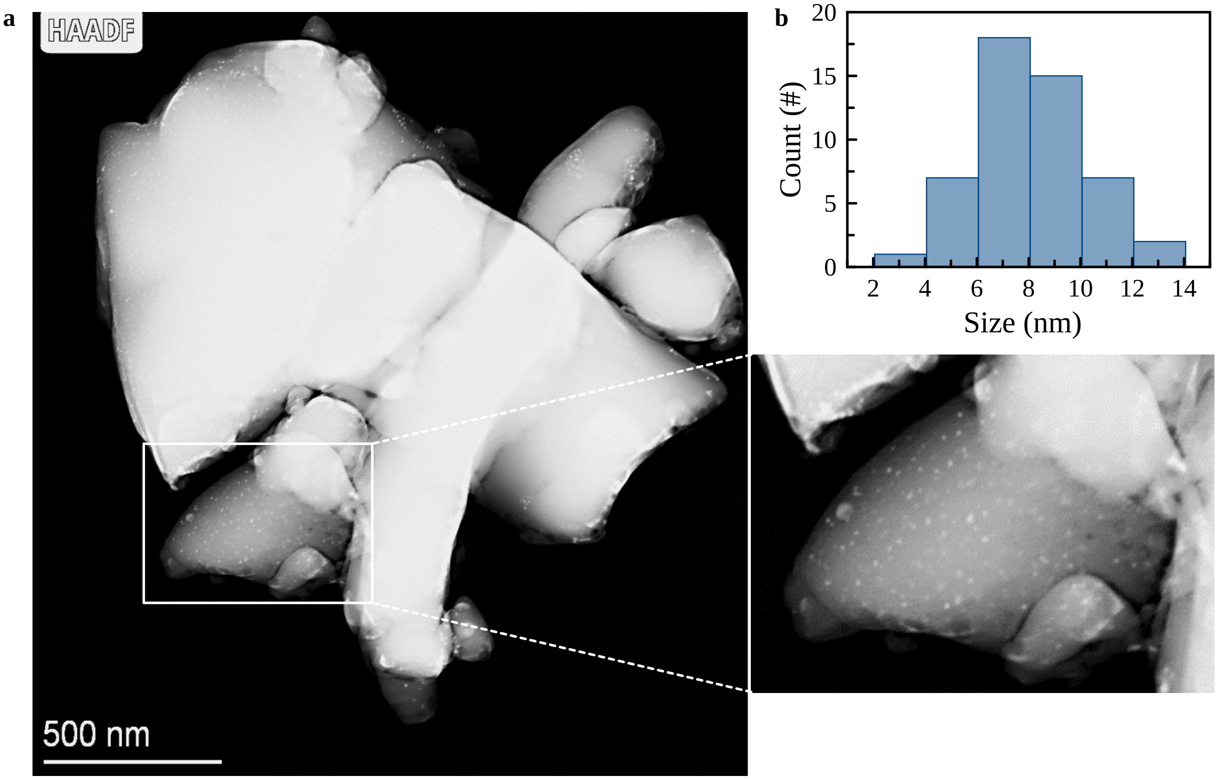

Supplementary Figure 14. **HAADF-STEM of NCs.** **a**, HAADF-STEM image for a small particle of the glass containing NCs. The last ones are seen as white dots on the image. See the zoomed in area on the right for a better visibility. **b**, Histogram of NC sizes for a zoomed in area, leading to the average NC size of 8 nm.

TEM measurements

We use the high-angle annular dark-field (HAADF) imaging in scanning transmission electron microscope to determine the size distribution of the NCs in the measured sample from the center of the glass plate. In the first run of measurements it was discovered, that the glass surface becomes strongly charged and obscures the measurements in TEM. In order to circumvent this problem, the sample was ground to powder in a agate mortar, placed in a carbon coated copper grid and only then scanned in STEM mode. Supplementary Figure 14(a) represents the HAADF image for one of the pieces, which allowed us to see the NCs in the glass. The zoomed in area allows one to see the NCs as white spots in the darker matrix. Using the calibrated ruler, we extract the sizes of this spots and create the histogram of the NC sizes, see Fig. 14(b). For evaluation of the NC size the software ImageJ was used. This measurement confirms the smaller size of the NCs in the central area of the glass plate with an average diameter of (8 ± 1) nm.

Evaluation of the sample composition

The position of the first XRD peak depends on the chlorine concentration (x) in $CsPb(Cl_xBr_{1-x})_3$. To determine the concentration of bromine and chlorine in the studied sample, we used XRD data and compared our data with the ICDD database [36]. $CsPbBr_3$ has a number 01-0729 in this database and $2\theta = 21.47°$, while $CsPbCl_3$ has a number 01-075-0411 and $2\theta = 22.4°$. These data points are shown in Fig. 15 by red dots. The linear dependence, according to Vegard's law, connecting these dots is an approximation and is used for evaluation of the chlorine concentration in the studied sample. So, for the sample under study, it leads to a composition of $CsPb(Cl_{0.56}Br_{0.44})_3$.

Crystal phase of NCs

Determining the crystal structure of the investigated nanocrystals is complex, and the data given in the literature are discordant. However, we would like to discuss the situation we are aware of:

1) To start, for bulk crystals, it is known (experimentally and supported theoretically) that for $CsPbX_3$ (X = I, Br, Cl, and mixtures) phase transitions occur above or close to room temperature from the cubic ($T \gtrsim 300$ K) to orthorhombic ($T \lesssim 300$ K) phase. However, certain aspects, like polycrystalline to single crystal differences, phase

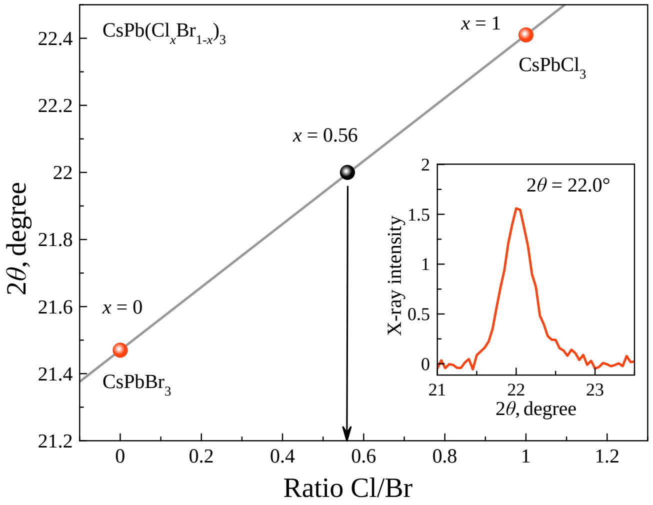

Supplementary Figure 15. **Determination of the Cl concentration.** The red dots show data from a database linking the diffraction angle to the chlorine concentration (x) in the $CsPb(Cl_xBr_{1-x})_3$ composite. The gray line connects these points. Black dot marks the sample under study. Insert demonstrates the first XRD peak position for the sample from the edge of the plate.

transition hysteresis protocols, etc., are still a matter of recent studies, though the determination started half century ago, see Refs. [37–42]). Note, pure $CsPbI_3$ is known to be unstable, so there are fewer reports on this type of crystal.

2) The situation becomes more scattered for nanocrystals. One of the main origins resulting in phase transitions of $CsPbX_3$, a tolerance factor $t < 1$ $(0.85 - 0.87)$, gets influenced by a rising importance of the crystal surface. Thus the size, shape, and host matrix (e.g., surface passivisation due to ligands, interface strain, etc.) need to be considered. To highlight a few reports, according to Ref. [43] the phase of small (5 nm) $CsPbBr_3$ NCs was observed to be cubic, while a mixture of cubic and orthorhombic fractions was present for larger NCs (10 nm) at room temperatures. This situation is also confirmed by Ref. [44] for $8 - 10$ nm cuboids, but in contrast to an observed orthorhombic phase for NC of the similar group in Ref. [45] gained with a different method. Further reports are present for nanoporous scaffolds, see Refs. [46, 47], colloids with ligands, see Refs. [48–51], and NCs in glass matrices, see Refs. [52–56]. Moreover, the identification of the proper phase is experimentally non-trivial as markers distinguishing between the phases present in the powder XRD spectra only slightly differ in their peak angle and may merge due to the drastic peak broadening present for NCs, similar to measurements in Supplementary Note 11. However, the higher stability of the cubic phase for nanocrystals is suggested to lower the respective phase transition temperature [57].

To summarize, for the studied $CsPb(Cl_{0.56}Br_{0.44})_3$ NCs, the crystal phase at room temperature is not unambiguously determinable to be either orthorhombic or cubic due to the low signal-to-noise ratio in the experiment and existing literature data. However, for most lead halide perovskite structures, a phase transition to the orthorhombic phase is observed sooner or later, with a lowering of the temperature. We may conclude that at the cryogenic temperatures of 1.6 to 5 K, at which we perform most of the experiments, the NCs phase is orthorhombic or less symmetric.

Finally, the presented theoretical approach for describing the SML effect is universal and applicable to different phases of the NCs. The phase would only define the coefficients $\sin\xi$ and $\cos\xi$ in the Eqs. (13-15), and would equally change the coefficients S_a and S_b, which would not affect the extracted spin coherence time T_2. Therefore, T_2 can also be determined from the calculation using the cubic phase of the NCs.

Phase segregation effect of NCs

Phase segregation, e.g. light induced, is a well known effect in mixed halide lead perovskites which efficiency depends on the perovskite composition. In general, for the classes of hybrid organic inorganic and all-inorganic crystals the effect differs. The hybrid organic inorganic perovskites, with the organic cations methylammonium and formamidinium, are less stable than those all-inorganic ones, with a cesium cation [58–60]. Therefore, cesium is often used to stabilize the phase of hybrid organic inorganic crystals. Within the class of purely inorganic crystals, the stability also shows a dependency on the composition of the triple solution of the anion. For example, crystals with a triple solution of bromine and iodine show phase segregation, which becomes less pronounced for crystals with a mixture of bromine and chlorine [60, 61]. This stability is further enhanced by the nanostructuring of the crystals [59]. So, for the $CsPb(Cl_{0.56}Br_{0.44})_3$ sample the phase segregation should play an insignificant role as compared to other metal halide perovskites.

In order to demonstrate the absence of a phase segregation effect in the studied sample, we performed additional

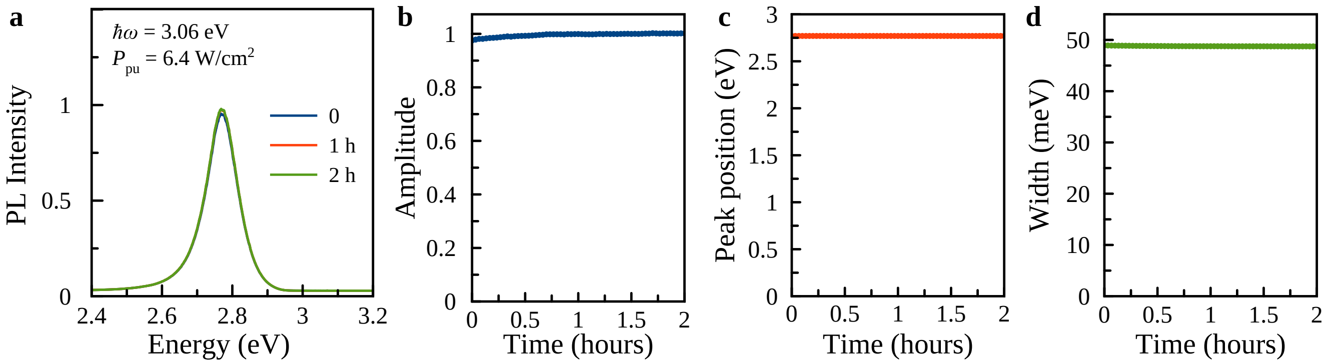

Supplementary Figure 16. **Phase segregation resistivity of NCs in glass.** **a**, PL measurements with three different time intervals of illumination at room temperature. The parameters of the PL peak over time: **b**, the peak amplitude, **c**, the peak position, and **d**, the peak width.

photoluminescence measurements at room temperature. The PL spectrum measured instantaneously after the start of excitation coincides with the spectrum detected after one and two hours of excitation, as shown in Fig. 16a. Non-resonant continuous wave laser excitation with optical energy 3.06 eV was used for the PL measurements. The light from this laser was focused into a large spot with a diameter of 100 μm, with the purpose to reproduce the conditions for the pump-probe experiments. To clearly show the absence of the phase segregation effect under such experimental conditions, we plotted the dependence of the PL peak amplitude Fig. 16b, position Fig. 16c, and width Fig. 16d as a function of the duration of illumination from zero to two hours. As one can see, all parameters remain constant in a given time range with very high accuracy of the measured value.

In conclusion, we would like to emphasize that the power density used in the experiments is too low to produce a phase segregation effect. The SML effect is investigated at the temperature of liquid helium in order to avoid local heating of the sample by the laser light. Additionally, we checked that the optical spectrum of PL at room temperature does not change any of the characteristics during continuous excitation for 2 hours.

SUPPLEMENTARY REFERENCES

[1] Kirstein, E., Yakovlev, D. R., Glazov, M. M., Evers, E., Zhukov, E. A., Belykh, V. V., Kopteva, N. E., Kudlacik, D., Nazarenko, O., Dirin, D. N., Kovalenko, M. V. and Bayer, M. Lead-dominated hyperfine interaction impacting the carrier spin dynamics in halide perovskites. *Advanced Materials* **34**, 2105263 (2022).

[2] Ivchenko, E. L. *Optical Spectroscopy of Semiconductor Nanostructures* (Alpha Science, Harrow, UK, 2005).

[3] Glazov, M. M. *Electron & Nuclear Spin Dynamics in Semiconductor Nanostructures* (Oxford University Press, Oxford, UK, 2018).

[4] Grigoryev, P. S., Belykh, V. V., Yakovlev, D. R., Lhuillier, E. and Bayer M. Coherent spin dynamics of electrons and holes in $CsPbBr_3$ colloidal nanocrystals. *Nano Lett.* **21**, 8481–8487 (2021).

[5] Canneson, D., Shornikova, E. V., Yakovlev, D. R., Rogge, T., Mitioglu, A. A., Ballottin, M. V., Christianen, P. C. M., Lhuillier, E., Bayer, M. and Biadala, L. Negatively charged and dark excitons in $CsPbBr_3$ perovskite nanocrystals revealed by high magnetic fields. *Nano Lett.* **17**, 6177–6183 (2017).

[6] Efros, Al. L. in *Semiconductor and Metal Nanocrystals: Synthesis and Electronic and Optical Properties* (ed. Klimov, V. I.) Chapter 3, pp. 103–141 (Dekker, New York, 2003).

[7] Kirstein, E., Yakovlev, D. R., Glazov, M. M., Zhukov, E. A., Kudlacik, D., Kalitukha, I. V., Sapega, V. F., Dimitriev, G. S., Semina, M. A., Nestoklon, M. O., Ivchenko, E. L., Kopteva, N. E., Dirin, D. N., Nazarenko, O., Kovalenko, M. V., Baumann, A., Höcker, J., Dyakonov, V. and Bayer, M. The Landé factors of electrons and holes in lead halide perovskites: universal dependence on the band gap. *Nature Commun.* **13**, 3062 (2022).

[8] Kalevich, V. K. and Korenev, V. L. Optical polarization of nuclei and ODNMR in GaAs/AlGaAs quantum wells. *Appl. Magn. Reson.* **2**, 397 (1991).

[9] Yugova, I. A., Glazov, M. M., Yakovlev, D. R., Sokolova, A. A. and Bayer, M. Coherent spin dynamics of electrons and holes in semiconductor quantum wells and quantum dots under periodical optical excitation: Resonant spin amplification versus spin mode locking. *Phys. Rev. B* **85**, 125304 (2012).

[10] Hernandez, F. G. G., Greilich, A., Brito, F., Wiemann, M., Yakovlev, D. R., Reuter, D., Wieck, A. D. and Bayer, M. Temperature-induced spin-coherence dissipation in quantum dots. *Phys. Rev. B* **78**, 041303 (2008).

[11] Varwig, S., René, A., Greilich, A., Yakovlev, D. R., Reuter, D., Wieck, A. D. and Bayer, M. Temperature dependence of hole spin coherence in (In,Ga)As quantum dots measured by mode-locking and echo techniques. *Phys. Rev. B* **87**, 115307 (2013).

[12] Varwig, S., Schwan, A., Barmscheid, D., Müller, C., Greilich, A., Yugova, I. A., Yakovlev, D. R., Reuter, D., Wieck, A. D. and Bayer, M. Hole spin precession in a (In,Ga)As quantum dot ensemble: From resonant spin amplification to spin mode locking. *Phys. Rev. B* **86**, 075321 (2012).

[13] Greilich, A., Oulton, R., Zhukov, E. A., Yugova, I. A., Yakovlev, D. R., Bayer, M., Shabaev, A., Efros, Al. L., Merkulov, I. A., Stavarache, V., Reuter, D. and Wieck, A. Optical control of spin coherence in singly charged (In,Ga)As/GaAs quantum dots. *Phys. Rev. Lett.* **96**, 227401 (2006).

[14] Heisterkamp, F., Zhukov, E. A., Greilich, A., Yakovlev, D. R., Korenev, V. L., Pawlis, A. and Bayer, M. Longitudinal and transverse spin dynamics of donor-bound electrons in fluorine-doped ZnSe: Spin inertia versus Hanle effect. *Phys. Rev. B* **91**, 235432 (2015).

[15] Smirnov, D. S., Zhukov, E. A., Kirstein, E., Yakovlev, D. R., Reuter, D., Wieck, A. D., Bayer, M., Greilich, A. and Glazov, M. M. Theory of spin inertia in singly charged quantum dots. *Phys. Rev. B* **98**, 125306 (2018).

[16] Mikhailov, A. V., Belykh, V. V., Yakovlev, D. R., Grigoryev, P. S., Reithmaier, J. P., Benyoucef, M. and Bayer, M. Electron and hole spin relaxation in InP-based self-assembled quantum dots emitting at telecom wavelengths. *Phys. Rev. B* **98**, 205306 (2018).

[17] Nestoklon, M. O. Tight-binding description of inorganic lead halide perovskites in cubic phase. *Computational Materials Science* **196**, 110535 (2021).

[18] Zhukov, E. A., Yakovlev, D. R., Bayer, M., Glazov, M. M., Ivchenko, E. L., Karczewski, G., Wojtowicz, T., and Kossut, J. Spin coherence of a two-dimensional electron gas induced by resonant excitation of trions and excitons in CdTe/(Cd,Mg)Te quantum wells. *Phys. Rev. B* **76**, 205310 (2007).

[19] Yakovlev, D. R. and Bayer, M. Coherent spin dynamics of carriers, Chapter 6 in *Spin Physics in Semiconductors* (ed. Dyakonov, M. I.) pp. 155–206 (Springer International Publishing AG, 2017).
[20] Bir, G. L. and Pikus, G. E. *Symmetry and Strain-Induced Effects in Semiconductors.* (Nauka, Moscow, 1972; Wiley, New York, 1975).
[21] Landau, L. D. and Lifshitz, E. M. *Quantum Mechanics: Non-Relativistic Theory.* (Pergamon Press, Oxford, 2006).
[22] Yugova, I. A., Glazov, M. M., Ivchenko, E. L. and Efros, Al. L. Pump-probe Faraday rotation and ellipticity in an ensemble of singly charged quantum dots. *Phys. Rev. B.* **80**, 104436 (2009).
[23] Greilich, A., Shabaev, A., Yakovlev, D. R., Efros, Al. L., Yugova, I. A., Reuter, D., Wieck, A. D. and Bayer, M. Nuclei-induced frequency focusing of electron spin coherence. *Science* **317**, 1896–1899 (2007).
[24] Kikkawa, J. M. and Awschalom, D. D. Resonant Spin Amplification in n-Type GaAs. *Phys. Rev. Lett.* **80**, 4313 (1998).
[25] Semiconductor Spintronics and Quantum Computation, edited by D. D. Awschalom, D. Loss, and N. Samarth (Springer, Heidelberg, 2002).
[26] Carter, S. G., Shabaev, A., Economou, Sophia E., Kennedy, T. A., Bracker, A. S. and Reinecke, T. L. Directing nuclear spin flips in InAs quantum dots using detuned optical pulse trains. *Phys. Rev. Lett.* **102**, 167403 (2009).
[27] Korenev V. L. Multiple stable states of a periodically driven electron spin in a quantum dot using circularly polarized light. *Phys. Rev. B* **83**, 235429 (2011).
[28] Glazov, M. M., Yugova, I. A. and Efros, Al. L. Electron spin synchronization induced by optical nuclear magnetic resonance feedback. *Phys. Rev. B* **85**, 041303(R) (2012).
[29] Beugeling, W., Uhrig, G. S. and Anders, F. B. Quantum model for mode locking in pulsed semiconductor quantum dots, *Phys. Rev. B* **94**, 245308 (2016).
[30] Jäschke, N., Fischer, A., Evers, E., Belykh, V. V., Greilich, A, Bayer, M. and Anders, F. B. Nonequilibrium nuclear spin distribution function in quantum dots subject to periodic pulses. *Phys. Rev. B* **96**, 205419 (2017).
[31] Zhukov, E. A., Kirstein, E., Kopteva, N. E., Heisterkamp, F., Yugova, I. A., Korenev, V. L., Yakovlev, D. R., Pawlis, A., Bayer, M. and Greilich, A. Discretization of the total magnetic field by the nuclear spin bath in fluorine-doped ZnSe. *Nat. Commun.* **9**, 1941 (2018).
[32] Evers, E., Kopteva, N. E., Yugova, I. A., Yakovlev, D. R., Bayer, M. and Greilich, A. Suppression of nuclear spin fluctuations in an InGaAs quantum dot ensemble by GHz-pulsed optical excitation. *npj Quantum Information* **7**, 60 (2021).
[33] Evers, E., Kopteva, N. E., Yugova, I. A., Yakovlev, D. R., Bayer, M. and Greilich, A. Shielding of external magnetic field by dynamic nuclear polarization in (In,Ga)As quantum dots. *Phys. Rev. B* **104**, 075302 (2021).
[34] Smirnov, D. S. Spin noise of localized electrons interacting with optically cooled nuclei, *Phys. Rev. B* **91**, 205301 (2015).
[35] Kolobkova, E. V., Kuznetsova, M. S. and Nikonorov, N. V. Perovskite $CsPbX_3$ (X=Cl, Br, I) nanocrystals in fluorophosphate glasses. *J. Non-Cryst. Solids* **563**, 120811 (2021).
[36] https://www.icdd.com
[37] Møller, C. K. Crystal Structure and Photoconductivity of Cæsium Plumbohalides. *Nature* **182**, 1436–1436 (1958).
[38] Fujii, Y., Hoshino, S., Yamada, Y. and Shirane, G. Neutron-scattering study on phase transitions of $CsPbCl_3$. *Phys. Rev. B* **9**, 4549–4559 (1974).
[39] Hirotsu, S., Harada, J., Iizumi, M. and Gesi, K. Structural Phase Transitions in CsPbBr3. *J. Phys. Soc. Jpn.* **37**, 1393–1398 (1974).
[40] Aleksandrov, K. S. and BartolomÉ, J. Structural distortions in families of perovskite-like crystals. *Phase Transitions* **74**, 255–335 (2001).
[41] Steele, J. A. et al. Phase Transitions and Anion Exchange in All-Inorganic Halide Perovskites. *Acc. Mater. Res.* **1**, 3–15 (2020).
[42] Liu, S. Z., DeFilippo, A. R., Balasubramanian, M., Liu, Z. X., Wang, S. G., Chen, Y., Chariton, S., Prakapenka, V., Luo, X. P., Zhao, L. Y., San Martin, J., Lin, Y. X., Yan, Y., Ghose, S. K. and Tyson, T. A. High-resolution in-situ synchrotron X-ray studies of inorganic perovskite $CsPbBr_3$: New symmetry assignments and structural phase transitions, *Advanced Science* **8**, 2003046 (2021).
[43] Brennan, M. C., Kuno, M. and Rouvimov, S. Crystal structure of individual $CsPbBr_3$ perovskite nanocubes, *Inorg. Chem.* **58**, 1555 (2019).
[44] Nedelcu, G., Protesescu, L., Yakunin, S., Bodnarchuk, M.I., Grotevent, M.J., Kovalenko, M. V. Fast anion-exchange in highly luminescent nanocrystals of cesium lead halide perovskites ($CsPbX_3$, X = Cl, Br, I), *Nano Lett.* **15**, 5635 (2015).
[45] Protesescu, L., Yakunin, S., Nazarenko, O., Dirin, D. N., and Kovalenko, M. V. Low-cost synthesis of highly luminescent colloidal lead halide perovskite nanocrystals by wet ball milling, *ACS Appl. Nano Mater.* **1**, 1300 (2018).
[46] Ma, S. et al. Strain-Mediated Phase Stabilization: A New Strategy for Ultrastable α-$CsPbI_3$ Perovskite by Nanoconfined Growth. *Small* **15**, 1900219 (2019).
[47] Kong, X., Shayan, K., Hua, S., Strauf, S. and Lee, S. S. Complete Suppression of Detrimental Polymorph Transitions in All-Inorganic Perovskites via Nanoconfinement. *ACS Appl. Energy Mater.* **2**, 2948–2955 (2019).
[48] Protesescu, L. et al. Nanocrystals of Cesium Lead Halide Perovskites ($CsPbX_3$, X = Cl, Br, and I): Novel Optoelectronic Materials Showing Bright Emission with Wide Color Gamut. *Nano Lett.* **15**, 3692–3696 (2015).
[49] Bodnarchuk, M. I. et al. Rationalizing and Controlling the Surface Structure and Electronic Passivation of Cesium Lead Halide Nanocrystals. *ACS Energy Lett.* **4**, 63–74 (2019).
[50] Fanizza, E. et al. Post-synthesis phase and shape evolution of $CsPbBr_3$ colloidal nanocrystals: The role of ligands. *Nano Res.* **12**, 1155–1166 (2019).
[51] McGrath, F., Ghorpade, U. V. and Ryan, K. M. Synthesis and dimensional control of $CsPbBr_3$ perovskite nanocrystals using phosphorous based ligands. *J. Chem. Phys.* **152**, 174702 (2020).

[52] Aryal, P. et al. Rapid and convenient crystallization of quantum dot $CsPbBr_3$ inside a phosphate glass matrix. *J. Alloys Compd* **866**, 158974 (2021).
[53] Liu, S. et al. Precipitation and tunable emission of cesium lead halide perovskites ($CsPbX_3$, X = Br, I) QDs in borosilicate glass. *Ceram. Int.* **44**, 4496–4499 (2018).
[54] Chen, D. et al. Promoting photoluminescence quantum yields of glass-stabilized $CsPbX_3$ (X = Cl, Br, I) perovskite quantum dots through fluorine doping. *Nanoscale* **11**, 17216–17221 (2019).
[55] Ye, Y. et al. Highly Luminescent Cesium Lead Halide Perovskite Nanocrystals Stabilized in Glasses for Light-Emitting Applications. *Adv. Opt. Mater.* **7**, 1801663 (2019).
[56] Xu, Y., Zhao, X., Xia, M. and Zhang, X. Perovskite nanocrystal doped all-inorganic glass for X-ray scintillators. *J. Mater. Chem. C* **9**, 5452–5459 (2021).
[57] Alaei, A., Circelli, A., Yuan, Y., Yang, Y. and Lee, S. S. Polymorphism in metal halide perovskites. *Mater. Adv.* **2**, 47–63 (2021).
[58] Beal, R. E. et al. Structural Origins of Light-Induced Phase Segregation in Organic-Inorganic Halide Perovskite Photovoltaic Materials. *Matter* 2, 207–219 (2020).
[59] Knight, A. J. and Herz, L. M. Preventing phase segregation in mixed-halide perovskites: a perspective. *Energy Environ. Sci.* **13**, 2024–2046 (2020).
[60] Tian, L., Xue, J., Wang, R. Halide segregation in mixed halide perovskites: visualization and mechanisms, *Electronics* **11**, 700 (2022).
[61] Ochsenbein, S. T., Krieg, F., Shynkarenko, Y., Rainó, G. and Kovalenko, M. V. Engineering Color-Stable Blue Light-Emitting Diodes with Lead Halide Perovskite Nanocrystals. *ACS Appl. Mater. Interfaces* **11**, 21655–21660 (2019).
